# Identification of Potential Immune Checkpoint Inhibitor Targets in Gliomas via Bioinformatic Analyses

**DOI:** 10.1155/2022/1734847

**Published:** 2022-02-14

**Authors:** Mengmeng Ding, Yong-an Li, Zhimin Lu, Guoxin Hou

**Affiliations:** ^1^Department of Neurology, Xiangcheng People's Hospital, Suzhou, China; ^2^Department of Outpatient, The First Hospital of Jiaxing, Affiliated Hospital of Jiaxing University, Jiaxing, Zhejiang, China; ^3^Department of Oncology, The First Hospital of Jiaxing, Affiliated Hospital of Jiaxing University, Jiaxing, Zhejiang, China

## Abstract

**Background:**

Glioma is a common tumor originating from the glial cells of the brain. Immune checkpoint inhibitors can potentially be used to treat gliomas, although no drug is currently approved.

**Methods:**

The expression levels of the immune checkpoint genes in glioma and normal tissues, and their correlation with the IDH mutation status and complete 1p/19q codeletion, were analyzed using The Cancer Genome Atlas (TCGA) and the Chinese Glioma Genome Atlas (CGGA) databases. Survival analyses were conducted using the CGGA database. Protein-protein interaction and functional enrichment analyses were performed via the STRING database using GO, KEGG, and Reactome pathways. The correlation between the immune checkpoints and the immune cell infiltration was determined using the TISIDB and TIMER databases.

**Results:**

HAVCR2 was overexpressed in the gliomas compared to normal brain tissues, as well as in the high-grade glioma patients and significantly downregulated in IDH mutant or 1p/19q codeletion patients. Overexpression of HAVCR2 was associated with poor survival in tumor grades II, III, and IV and was the most correlated with immune infiltration of B and T cells.

**Conclusion:**

HAVCR2 can be a potential therapeutic target for cancer immunotherapy for glioma patients.

## 1. Introduction

Glioma is a common tumor that originates from the glial cells of the brain [1] and accounts for more than 30% of all brain tumors and 80% of the malignant tumors [2]. According to the World Health Organization (WHO) criteria [3], gliomas are classified into the following grades: (i) benign gliomas (grade I) with relatively low risk that can be removed by surgery depending on their location [4], (ii) low-grade gliomas (LGG) that consist of grades II and III and have highly variable clinical behavior [5], and (iii) glioblastomas (GBM, grade IV) that arise from LGGs and are the most aggressive type [6]. At the molecular level, gliomas are characterized by IDH mutations and complete deletion of chromosome 1p and 19q arms [7]. Patients with IDH mutation and complete 1p/19q codeletion generally have a better prognosis compared to the IDH wild-type patients, who might also have a different response to therapy [8].

Gliomas are currently diagnosed by MRI scans, CT scans, and tissue biopsies, and the treatment strategy typically includes surgery followed by chemotherapy and radiation. The survival duration of gliomas patients varies from months to years [4, 9], and the LGG patients have a median survival of more than 10 years and a 10-year survival rate of 47% [10–12]. The overall survival of high-grade GBM patients after diagnosis is around 12~15 months, and the 5-year survival rate is about 3~7% [13].

Immunotherapy is increasingly being considered as a potential treatment for gliomas, especially with immune checkpoint inhibitors (ICIs). The currently approved checkpoint inhibitors block PD-1/PD-L1 and CTLA4 and have been tested in multiple cancers, including breast cancer [14, 15], lung cancer [16, 17], and skin cancer [18, 19]. However, ICIs have achieved limited results against brain cancer including GBM and LGG, and only 10% of GBM patients benefit from immunotherapy [20]. Moreover, only a few studies have analyzed the expression of the immune checkpoints in brain cancer, and their roles have not been examined yet. Therefore, there is an urgent need to explore the landscape of the immune checkpoints in gliomas and their potential as targets for cancer immunotherapy. The identification of the most promising immune checkpoints can guide future clinical trials for gliomas. In this study, we systematically analyzed the expression levels of the eight immune checkpoint genes and their prognostic value in gliomas using integrative analyses. The gene expression levels were compared between glioma and normal brain tissues, as well as across different grades. Furthermore, the association between the expression of each immune checkpoint gene and IDH mutation, complete 1p/19q codeletion, overall survival, and tumor-immune infiltration was evaluated.

## 2. Materials and Methods

### 2.1. Data Source

The GBM and LGG datasets were retrieved from The Cancer Genome Atlas (TCGA) [5, 21] and the Chinese Glioma Genome Atlas (CGGA) [22] databases. A total of 207 normal and 163 GBM tumor samples and 207 normal and 518 LGG tumor samples, as well as the clinical information including age, gender, tumor grade, and survival rate of patients, were curated in TCGA. The CGGA contains brain tumor datasets of over 2000 samples from Chinese cohorts, including genomic sequencing data and matched clinical data. The eight immune checkpoint genes, including CD274, CTLA4, HAVCR2, LAG3, TIGIT, PDCD1, PDCD1LG2, and SIGLEC15, were manually collected from the literature.

### 2.2. Expression Analysis of the Immune Checkpoint Genes

The expression levels of the immune checkpoint genes in glioma and normal samples were analyzed using the GEPIA database (http://gepia.cancer-pku.cn/) [23, 24]. The association of each immune checkpoint gene with different WHO grades, IDH mutation status, and complete 1p/19q codeletion was analyzed using the CGGA database (http://www.cgga.org.cn/).

### 2.3. Survival Analyses

Overal survival (OS) of the high and low expression patient groups pertaining to each immune checkpoint gene was plotted by the Kaplan-Meier method using the CGGA database [22]. The median gene expression value was used to demarcate the high and low gene expression groups. The log-rank test was used to analyze differences in OS between the two groups. A *p* value < 0.05 was considered statistically significant.

### 2.4. Gene Function and Pathway Enrichment Analyses

The top 100 genes with the highest correlation to HAVCR2 obtained from the GEPIA database were functionally annotated by Gene Ontology (GO) [25, 26], KEGG [27–29], and Reactome [30, 31] pathway enrichment analyses. The Search Tool for the Retrieval of Interacting Genes (STRING, http://www.string-db.org/) database was used to analyze protein-protein interaction (PPI), gene function, and pathway enrichment analyses [32–34]. The top 5 terms based on the false discovery rate (FDR) were considered significantly enriched.

### 2.5. Correlation between the Abundance of Immune Checkpoint Genes and Tumor-Immune System Interactions

The correlation of immune checkpoint genes with tumor-infiltrating lymphocytes (TILs), immunomodulators (i.e., immunoinhibitors, immunostimulators, and MHC molecules), chemokines, and receptors was performed using the TISIDB database (http://cis.hku.hk/TISIDB/), an integrated repository portal for tumor-immune system interactions [35]. The top 3 interactions according to the Spearman correlation test were considered the most significant. The TIMER [36, 37] database (https://cistrome.shinyapps.io/timer/) was also used to analyze the association between the immune checkpoint genes and immune cell infiltration.

## 3. Results

### 3.1. Expression of the Immune Checkpoint Genes in Glioma and Normal Samples

**Figure 1:**
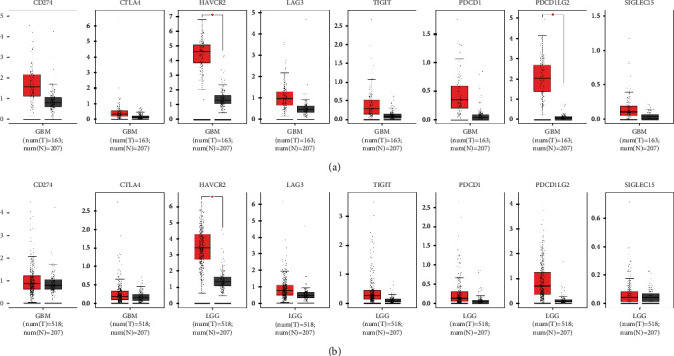
Expression profile of the immune checkpoint genes analyzed by GEPIA. (a) Expression of immune checkpoint genes in GBM tissues versus normal tissues. (b) Expression of immune checkpoint genes in LGG tissues versus normal tissues.

To determine whether the immune checkpoint genes were differentially expressed in the gliomas relative to normal brain tissues, we evaluated their expression levels using the GBM and LGG datasets in TCGA. All eight immune checkpoint genes were overexpressed in both GBM and LGG tumors (Figure 1), of which only HAVCR2 was significantly upregulated in both glioma types and PDCD1LG2 showed significant upregulation in GBM but not in LGG (*p* < 0.05).

### 3.2. The Expression of the Immune Checkpoint Genes Correlates with Age and Gender

We also analyzed whether the expression of the immune checkpoint genes was associated with age and gender using the CGGA database. The expression levels of CD274, CTLA4, HAVCR2, TIGIT, PDCD1, PDCD1LG2, and SIGLEC15 were significantly correlated to age, and the older patients expressed higher levels (Figure S1, *p* < 0.05). In contrast, none of the eight immune checkpoint genes was correlated to gender (Figure S1, *p* > 0.05).

### 3.3. Expression of the Immune Checkpoint Genes in Different Glioma Grades

**Figure 2:**
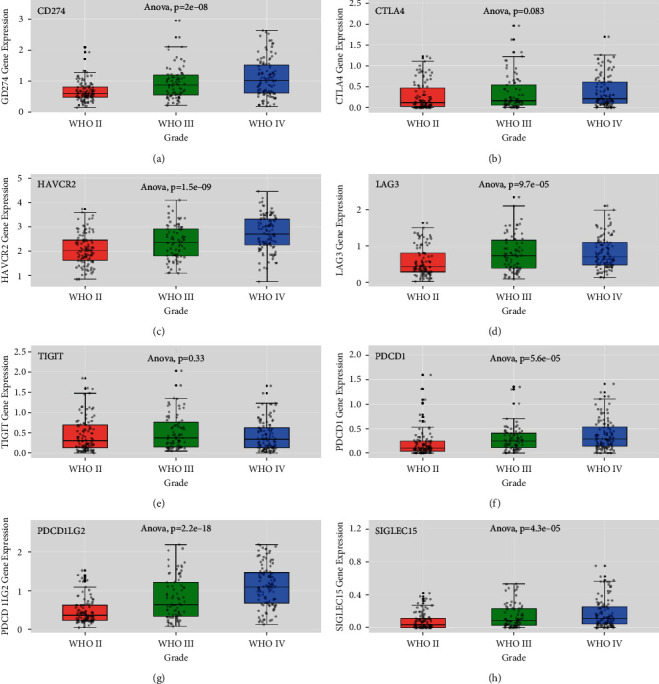
Expression of the immune checkpoint genes in different glioma grades. Expression of (a) CD274, (b) CTLA4, (c) HAVCR2, (d) LAG3, (e) TIGIT, (f) PDCD1, (g) PDCD1LG2, and (h) SIGLEC15 in gliomas according to WHO grade status in the CGGA databases.

Furthermore, we analyzed the association between the immune checkpoint genes and the different tumor grades using the CGGA database. The expression levels of all immune checkpoint genes were significantly correlated to the tumor grades, and higher gene expression accompanied higher tumor grade. The highest expression was detected in the WHO glioma grade IV tumors (CD274 *p* = 2.0e − 08, HAVCR2 *p* = 1.5e − 09, LAG3 *p* = 9.7e − 05, PDCD1 *p* = 5.6e − 05, PDCD1LG2 *p* = 2.2e − 18, and SIGLEC15 *p* = 4.3e − 05) (Figure 2).

### 3.4. Association between the Immune Checkpoint Gene Expression and IDH Mutation Status

**Figure 3:**
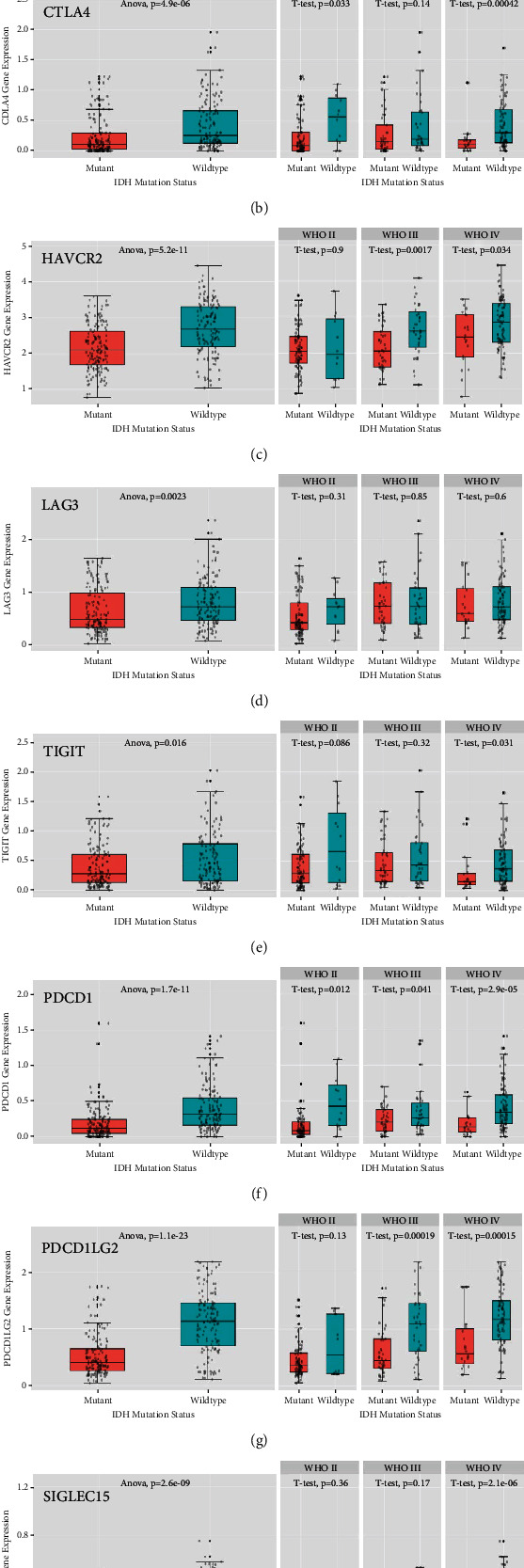
Association of IDH mutation status and immune checkpoint gene expression in gliomas in the CGGA databases: (a) CD274, (b) CTLA4, (c) HAVCR2, (d) LAG3, (e) TIGIT, (f) PDCD1, (g) PDCD1LG2, and (h) SIGLEC15.

Since IDH mutation is an important marker in glioma classification, we investigated its relationship with the expression of immune checkpoint genes using the CGGA database. The immune checkpoint genes were all highly expressed in IDH wild-type tumors compared to the mutant tumors (CD274 *p* = 8.4e − 16, CTLA4 *p* = 4.9e − 06, HAVCR2 *p* = 5.2e − 11, LAG3 *p* = 2.3e − 03, TIGIT *p* = 1.6e − 02, PDCD1 *p* = 1.7e − 11, PDCD1LG2 *p* = 1.1e − 23, and SIGLEC15 *p* = 2.6e − 09), and similar patterns were observed after stratifying patients according to tumor grade, except for LAG3 (CD274 WHO II *p* = 8.7e − 03, WHO III *p* = 2.3e − 02, and WHO IV *p* = 1.0e − 06; CTLA4 WHO II *p* = 3.3e − 02, WHO III *p* = 0.14, and WHO IV *p* = 4.2e − 04; HAVCR2 WHO II *p* = 0.9, WHO III *p* = 1.7e − 03, and WHO IV *p* = 3.4e − 02; LAG3 WHO II *p* = 0.31, WHO III *p* = 0.85, and WHO IV *p* = 0.6; TIGIT WHO II *p* = 8.6e − 02, WHO III *p* = 0.32, and WHO IV *p* = 3.1e − 02; PDCD1 WHO II *p* = 1.2e − 02, WHO III *p* = 4.1e − 02, and WHO IV *p* = 2.9e − 05; PDCD1LG2 WHO II *p* = 0.13, WHO III *p* = 1.9e − 04, and WHO IV *p* = 1.5e − 04; and SIGLEC15 WHO II *p* = 3.6e − 02, WHO III *p* = 0.17, and WHO IV *p* = 2.1e − 06) (Figure 3).

### 3.5. Association between the Immune Checkpoint Gene Expression and 1p/19q Codeletion Status

**Figure 4:**
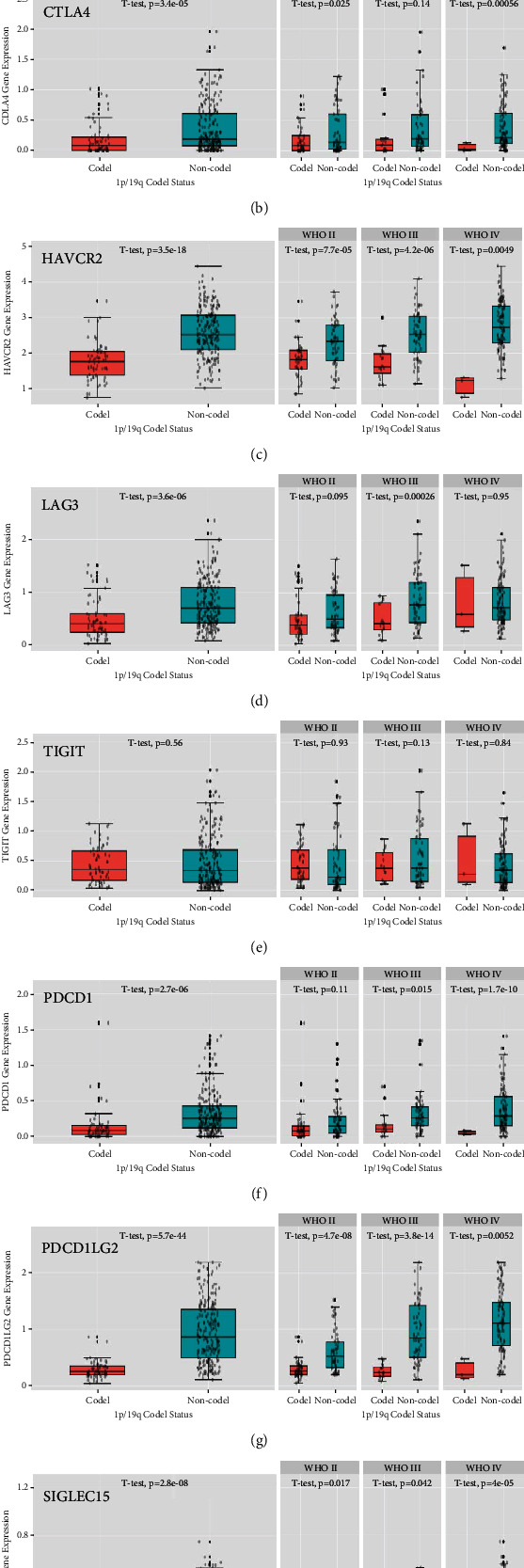
Association of 1p/19q codeletion and immune checkpoint gene expression in glioma in the CGGA databases: (a) CD274, (b) CTLA4, (c) HAVCR2, (d) LAG3, (e) TIGIT, (f) PDCD1, (g) PDCD1LG2, and (h) SIGLEC15.

Another significant marker glioma prognosis and response to therapy is the deletion of both the short arm of chromosome 1 and the long arm of chromosome 19 (1p/19q codeletion), which was analyzed using the CGGA database. Except for TIGIT, all immune checkpoint genes were significantly downregulated in 1p/19q codeletion patients (CD274 *p* = 2.5e − 21, CTLA4 *p* = 3.4e − 05, HAVCR2 *p* = 3.5e − 18, LAG3 *p* = 3.6e − 06, TIGIT *p* = 0.56, PDCD1 *p* = 2.7e − 06, PDCD1LG2 *p* = 5.7e − 44, and SIGLEC15 *p* = 2.8e − 08), even when patients were stratified according to tumor grade (CD274 WHO II *p* = 1.5e − 05, WHO III *p* = 8.0e − 06, and WHO IV *p* = 1.3e − 02; CTLA4 WHO II *p* = 2.5e − 02, WHO III *p* = 0.14, and WHO IV *p* = 5.6e − 04; HAVCR2 WHO II *p* = 7.7e − 05, WHO III *p* = 4.2e − 06, and WHO IV *p* = 4.9e − 03; LAG3 WHO II *p* = 9.5e − 02, WHO III *p* = 2.6e − 04, and WHO IV *p* = 0.95; TIGIT WHO II *p* = 0.93, WHO III *p* = 0.13, and WHO IV *p* = 0.84; PDCD1 WHO II *p* = 0.11, WHO III *p* = 1.5e − 02, and WHO IV *p* = 1.7e − 10; PDCD1LG2 WHO II *p* = 4.7e − 08, WHO III *p* = 3.8e − 14, and WHO IV *p* = 5.2e − 03; and SIGLEC15 WHO II *p* = 1.7e − 02, WHO III *p* = 4.2e − 02, and WHO IV *p* = 4.0e − 05) (Figure 4).

### 3.6. Survival Analysis of the Immune Checkpoint Genes

**Figure 5:**
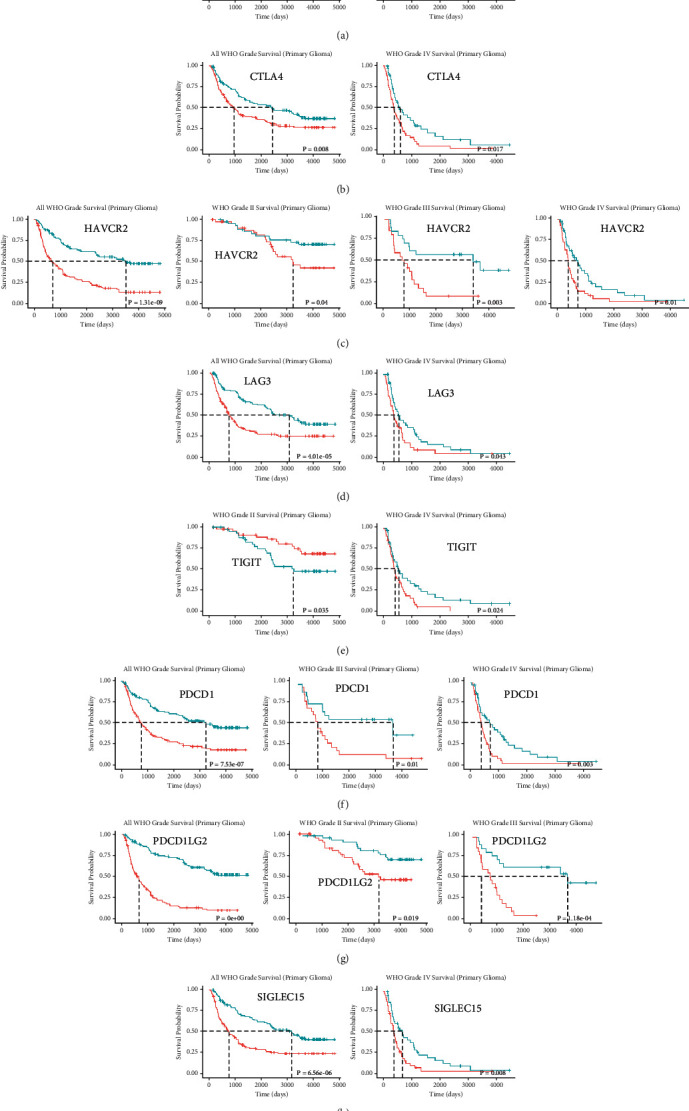
Kaplan-Meier curves showing overall survival of glioma patients stratified into the high and low expression groups of each immune checkpoint gene. (a) The prognostic effect of CD274 expression in all WHO grades and WHO grade IV. (b) The prognostic effect of CTLA4 expression in all WHO grade and WHO grade IV. (c) The prognostic effect of HAVCR2 expression in all WHO grades, WHO grade II, WHO grade III and WHO grade IV. (d) The prognostic effect of LAG3 expression in all WHO grades and WHO grade IV. (e) The prognostic effect of TIGIT expression in WHO grade II and WHO grade IV. (f) The prognostic effect of PDCD1 expression in all WHO grades, WHO grade III, and WHO grade IV. (g) The prognostic effect of PDCD1LG2 expression in all WHO grades, WHO grade II, and WHO grade III. (h) The prognostic effect of SIGLEC15 expression in all WHO grades and WHO grade IV.

By comparing the differences in OS between high and low gene expression groups using the CGGA database, we found that the expression levels of all immune checkpoint genes were significantly associated with poor survival. The overexpression of CD274 was related to poor survival in all WHO grades (*p* = 1.8e − 06) and WHO grade IV (*p* = 5.1e − 02) but not in WHO grades II and III (*p* = 0.242 and *p* = 0.109, respectively). Increased CTLA4 mRNA expression was correlated with significantly shorter survival for all WHO grades (*p* = 8.0e − 03) and WHO grade IV (*p* = 1.7e − 02) but not in WHO grades II and III (*p* = 0.819 and *p* = 0.963, respectively). Also, higher expression of LAG3 was associated with worse survival in all WHO grades (*p* = 4.01e − 05) and WHO grade IV (*p* = 4.3e − 02) but not in WHO grades II and III (*p* = 0.809 and *p* = 0.294, respectively). The overexpression of TIGIT mRNA was associated with favorable survival in WHO grade II (*p* = 3.5e − 02) and worse survival in WHO grade IV (*p* = 2.4e − 02) but had no effect on all WHO grades (*p* = 0.941) and WHO grade III (*p* = 0.134). Elevated PDCD1 mRNA expression was associated with worse survival for all WHO grades (*p* = 7.53e − 07), WHO grade III (*p* = 0.01), and WHO grade IV (*p* = 3.0e − 03) but not WHO grade II (*p* = 0.482). Elevated expression of PDCD1LG2 mRNA was also associated with shorter survival in all WHO grades (*p* < 0.001), WHO grade II (*p* = 1.9e − 02), and WHO grade III (*p* = 1.18e − 04) but not in WHO grade IV (*p* = 0.425). Moreover, the increased expression of SIGLEC15 predicted worse survival in all WHO grades (*p* = 6.56e − 06) and WHO grade IV (*p* = 8.0e − 03) but not in WHO grades II and III (*p* = 0.539 and *p* = 0.679, respectively). Also, only the overexpression of HAVCR2 led to poor survival in all WHO grades (*p* = 1.31e − 09), WHO grade II (*p* = 4.0e − 02), WHO grade III (*p* = 3.0e − 03), and WHO grade IV (*p* = 1.0e − 02) (Figure 5 and Figure S2).

### 3.7. Correlation of the Eight Immune Checkpoint Genes with Each Other

The correlation between the different immune checkpoint genes was analyzed using TIMER. In GBM, CD274 expression was positively correlated to HAVCR2, PDCD1LG2, and SIGLEC15, while that of CTLA4 correlated positively with HAVCR2, LAG3, TIGIT, PDCD1, PDCD1LG2, and SIGLEC15. In addition, the expression of HAVCR2 was positively correlated to CD274, CTLA4, TIGIT, PDCD1, PDCD1LG2, and SIGLEC15, and that of LAG3 was unrelated to CTLA4, TIGIT, PDCD1, and SIGLEC15. The expression of TIGIT had a positive correlation with CTLA4, HAVCR2, LAG3, PDCD1, PDCD1LG2, and SIGLEC15. PDCD1 expression was also positively correlated to CTLA4, HAVCR2, LAG3, TIGIT, PDCD1LG2, and SIGLEC15. The expression of PDCD1LG2 was positively correlated to CD274, CTLA4, HAVCR2, TIGIT, PDCD1, and SIGLEC15. In addition, SIGLEC15 expression was positively correlated to CD274, CTLA4, HAVCR2, LAG3, TIGIT, PDCD1c, and PDCD1LG2 (Figure S3A-B).

In LGG, the expression of CD274 was positively correlated to CTLA4, HAVCR2, TIGIT, PDCD1, PDCD1LG2, and SIGLEC15, and that of CTLA4 to CD274, HAVCR2, LAG3, TIGIT, PDCD1, PDCD1LG2, and SIGLEC15. The expression of HAVCR2 was positively correlated to CD274, CTLA4, LAG3, TIGIT, PDCD1, and PDCD1LG2, and LAG3 expression level was unrelated to CTLA4, HAVCR2, PDCD1, PDCD1LG2, and SIGLEC15. Moreover, TIGIT expression showed a positive correlation with CD274, CTLA4, HAVCR2, PDCD1, PDCD1LG2, and SIGLEC15, and PDCD1 expression was positively correlated to CD274, CTLA4, HAVCR2, LAG3, TIGIT, PDCD1LG2, and SIGLEC15. The expression of PDCD1LG2 was positively correlated to CD274, CTLA4, HAVCR2, LAG3, TIGIT, PDCD1, and PDCD1LG2. Finally, SIGLEC15 expression was positively correlated to CD274, CTLA4, LAG3, TIGIT, and PDCD1 (Figure S3C-D).

### 3.8. Gene Functions and Pathways Enrichment Analyses

Only HAVCR2 significantly upregulated in both GBM and LGG tumors (Figure 1), and its overexpression led to poor survival in all WHO grade, WHO grade II, WHO grade III, and WHO grade IV. Therefore, HAVCR2 was selected for further analyses. The top 100 genes most related to HAVCR2 obtained from the GEPIA database were used for GO and KEGG pathway enrichment analyses by using the STRING. The genes were mainly enriched in immune response, regulation of the immune response, leukocyte activation, and other biological processes (BP). The significant molecular functions (MF) included phosphotyrosine residue binding, lipid binding, and signaling receptor activity, and cell components (CC) such as plasma membrane, vesicle, and cytoplasmic vesicles were enriched. In addition, the B cell receptor signaling pathway, innate immune system, adaptive immune system, and signaling by interleukins were significantly enriched (Tables 1 and 2).

### 3.9. Correlation between the Abundance of HAVCR2 and Tumor-Immune System Interactions

**Figure 6:**
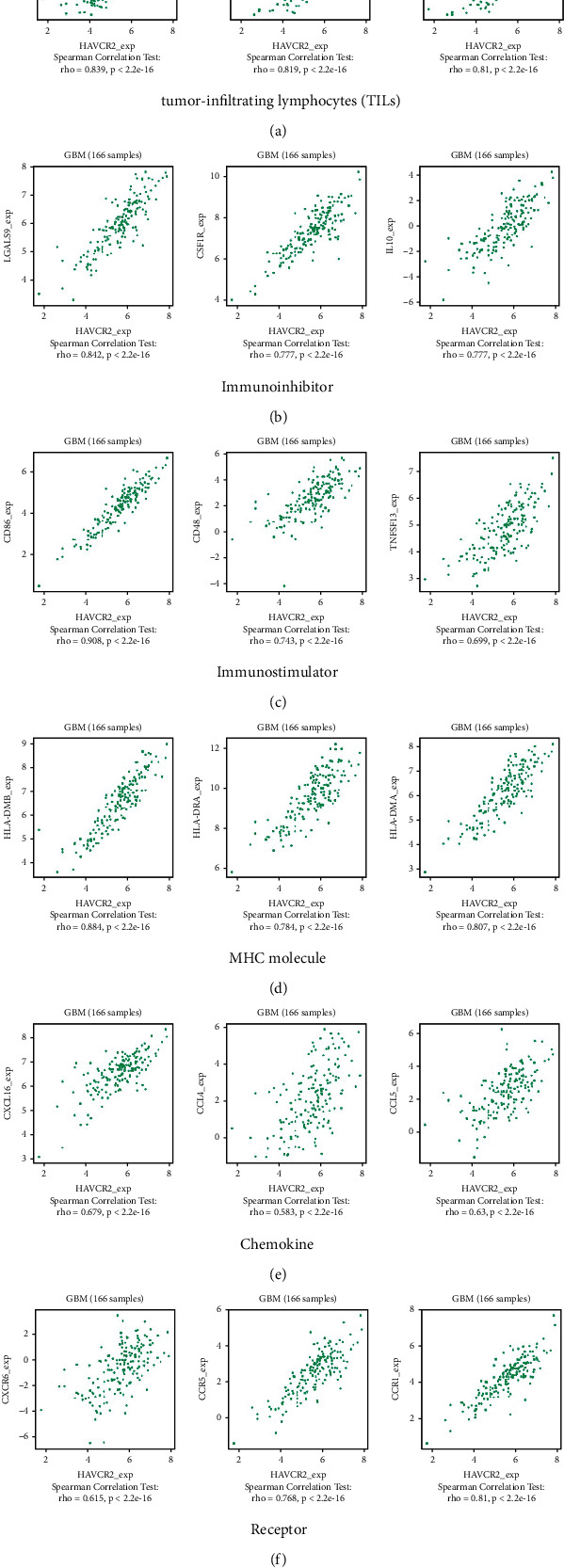
Correlation between HAVCR2 gene expression and tumor-immune system interactions in GBM. Correlation between HAVCR2 gene expression and (a) tumor-infiltrating lymphocytes (TILs), (b) immune inhibitors, (c) immune stimulators, (d) MHC molecules, (e) chemokines, and (f) receptors.

**Figure 7:**
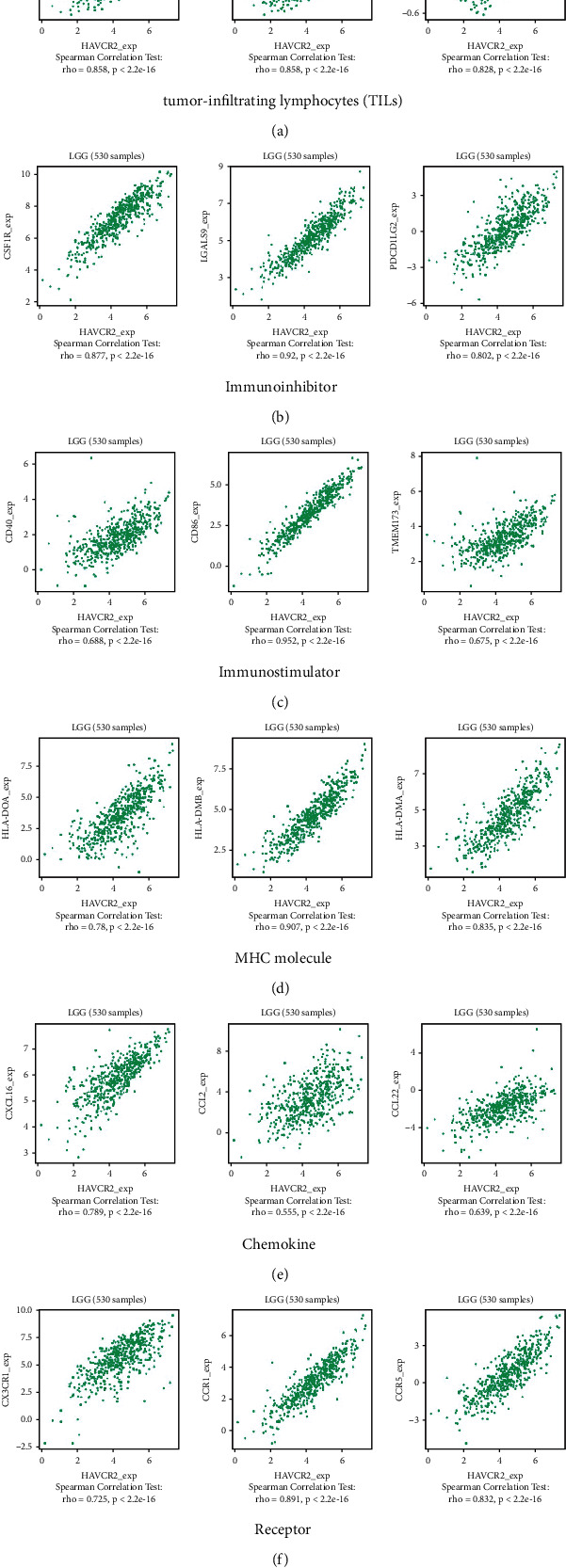
Correlation between HAVCR2 gene expression and tumor-immune system interactions in LGG. Correlation between HAVCR2 gene expression and (a) tumor-infiltrating lymphocytes (TIL2), (b) immune inhibitors, (c) immune stimulators, (d) MHC molecules, (e) chemokine, and (f) receptors.

We explored the correlation between HAVCR2 gene expression and TILs, as well as immunomodulators, chemokines, and receptors using the TISIDB database. In both GBM and LGG (Figure S4, Figures 6 and 7), HAVCR2 was positively correlated with MDSC, macrophage and Tfh, the immune inhibitors LGALS9 and CSF1R, the immune stimulator CD86, and MHC molecules including HLA-DMB and HLA-DMA. Likewise, CXCL16 chemokine and the CCR5 and CCR1 receptors were significantly and positively correlated with HAVCR2 gene expression. Moreover, HAVCR2 was also the most correlated gene with the immune infiltration of B and T cells as per TIMER (Figures S5 and S6).

## 4. Discussion

Despite continued efforts over past decades to develop new therapies for glioma, none has appreciably improved patient survival. Although immunotherapy has been successful against various cancers, immune checkpoint inhibitors have failed to increase the survival of advanced glioma patients [38]. Therefore, it is urgent to study immune checkpoints in gliomas and explore their potential as targets for cancer immunotherapy. In the present study, we systematically investigated the expression of immune checkpoint genes in gliomas and their prognostic value across tumor grades, OS, IDH mutation status, complete 1p/19q codeletion, and immune infiltration.

The immune checkpoint genes evaluated included CD274, also known as PD-L1, that encodes an immune inhibitory ligand expressed by hematopoietic and nonhematopoietic cells such as T cells and various tumor cells [39]. CTLA4, a member of the immunoglobulin superfamily, encodes a protein that transmits an inhibitory signal to T cells [40]. HAVCR2 or TIM3 is a cell surface marker expressed on CD8+ Th1 and CD4+ Th1 cells [41]. LAG3 belongs to the Ig superfamily and contains extracellular Ig-like domains [42]. TIGIT encodes a member of the poliovirus receptor family of immunoglobulin proteins and is expressed on several T cell classes [43]. PDCD1 or PD-1 is an immune inhibitory receptor expressed in activated T cells and is involved in the regulation of their functions [44]. PDCD1LG2, also known as PD-L2, is the other ligand for PD-1 [45]. In addition to the PD-1/PD-L1 pathway, SIGLEC15 is another important tumor-immune escape mechanism and represents a new kind of immune checkpoint inhibitor [46].

Although all of the above immune checkpoint genes were upregulated, only HAVCR2 and PDCD1LG2 were significantly overexpressed in gliomas compared to normal samples. HAVCR2 recruits immune cells and is positively correlated with the expression levels of CCL18, CXCL13, and CCL7, which can be used for predicting the prognosis of GBM patients [47]. Moreover, HAVCR2 levels are correlated with enhanced NK cell cytotoxicity and improved clinical outcomes in AML patients [48]. In addition, PDCD1LG2 overexpression is associated with poor prognosis in hepatocellular carcinoma patients [49]. Huang et al. found that advanced stage colon carcinoma patients with elevated tumor PD-L2 levels had a favorable 5-year OS compared to those with low PD-L2 levels [45]. Altogether, these findings suggested that glioma patients might benefit from HAVCR2- or PDCD1LG2-based immunotherapies.

Moreover, seven of the eight immune checkpoint genes were significantly related to age and tumor grades but not gender. Surprisingly, these immune checkpoint genes were almost significantly downregulated in IDH mutant or 1p/19q codeletion patients, even when patients were stratified according to tumor grade. Lin et al. recently reported that the expression of immune checkpoint genes decreased gradually from IDH wild to IDH mut or 1p/19q codeletion types [50].

Furthermore, HAVCR2 overexpression was significantly related to poor survival in all tumor grades, as well as grades II, III, and IV. The top 100 genes with the highest correlation to HAVCR2 were significantly enriched in immune response, regulation of the immune response, and leukocyte activation GO terms, as well as B cell receptor signaling pathway, innate immune system, adaptive immune system, and signaling by interleukins pathways. Furthermore, HAVCR2 was most positively correlated with MDSC, macrophage and Tfh TILs, LGALS9 and CSF1R immune inhibitors, CD86 immune stimulator, and MHC molecules such as HLA-DMB and HLA-DMA. Similarly, CXCL16 and the chemokine receptors CCR5 and CCR1 were significantly and positively correlated with HAVCR2 gene expression. Moreover, HAVCR2 was the most correlated gene with the infiltration of B and T cells, suggesting that HAVCR2 is a potential target for cancer immunotherapy.

HAVCR2 is overexpressed in the TILs in gastric [51], lung [52], and head and neck cancers [53]. Furthermore, Wu et al. reported that a high HAVCR2 expression potended worse outcome in LGG [54]. Recently, HAVCR2 overexpression was associated with T cell exhaustion in multiple cancers [55]. The high expression of CD276/HAVCR2 predicts an adverse GBM immune subtype and is closely related to the epithelial-mesenchymal transition [56]. These studies are consistent with our current findings, indicating that HAVCR2 is an important marker for gliomas and a potential target for cancer immunotherapy.

To summarize, HAVCR2 is significantly associated with the glioma grade, overall survival, IDH mutation status, complete 1p/19q codeletion, and infiltration of immune cells. Hence, HAVCR2 is a potential biomarker for the diagnosis, treatment, and prognosis of gliomas and should be developed further as a therapeutic target for antiglioma immunotherapies.

## Figures and Tables

**Table 1:** Top 5 GO items related to proteins involved in the network.

GO term	Description	False discovery rate
Biological process (BP)		
GO:0002376	Immune system process	1.95E-35
GO:0006955	Immune response	1.95E-35
GO:0002682	Regulation of immune system process	4.87E-30
GO:0050776	Regulation of immune response	1.3E-29
GO:0045321	Leukocyte activation	4.28E-28
Molecular function (MF)		
GO:0001784	Phosphotyrosine residue binding	0.00061
GO:0008289	Lipid binding	0.00061
GO:0038023	Signaling receptor activity	0.00061
GO:0051219	Phosphoprotein binding	0.00061
GO:0005543	Phospholipid binding	0.0014
Cellular component (CC)		
GO:0005886	Plasma membrane	8.58E-17
GO:0031982	Vesicle	1.86E-12
GO:0031410	Cytoplasmic vesicle	1.94E-12
GO:0030141	Secretory granule	2.64E-12
GO:0030667	Secretory granule membrane	1.12E-12

**Table 2:** Top 5 KEGG and Reactome pathways related to proteins involved in the network.

Pathway term	Description	False discovery rate
KEGG pathways		
hsa04380	Osteoclast differentiation	8.45E-09
hsa05150	Staphylococcus aureus infection	2.94E-08
hsa04662	B cell receptor signaling pathway	3.19E-06
hsa04664	Fc epsilon RI signaling pathway	3.19E-06
hsa05140	Leishmaniasis	3.19E-06
Reactome pathways		
HSA-168256	Immune system	1.93E-33
HSA-168249	Innate immune system	1.01E-28
HSA-1280218	Adaptive immune system	5.13E-15
HSA-6798695	Neutrophil degranulation	2.57E-14
HSA-449147	Signaling by interleukins	8.30E-11

## Data Availability

The [DATA TYPE] data used to support the findings of this study are included within the supplementary information.

## Supplementary Materials

Supplementary Materials Figure S1: the expression of immune checkpoint genes correlates with age and gender: (A) CD274, (B) CTLA4, (C) HAVCR2, (D) LAG3, (E) TIGIT, (F) PDCD1, (G) PDCD1LG2, and (H) SIGLEC15. Figure S2: Kaplan-Meier overall survival curves of the immune checkpoint genes in glioma: (A) CD274, (B) CTLA4, (C) LAG3, (D) TIGIT, (E) PDCD1, (F) PDCD1LG2, and (G) SIGLEC15. Figure S3: Correlation between the eight immune checkpoint genes: (A-B) GBM and (C-D) LGG. Figure S4: heatmaps showing the correlation between HAVCR2 gene expression and tumor-immune system interactions across all cancers in TCGA. Correlation between HAVCR2 gene expression and (A) tumor-infiltrating lymphocytes (TILs), (B) immune inhibitors, (C) immune stimulators, (D) MHC molecules, (E) chemokines, and (F) receptors. Figure S5: correlation of immune checkpoint gene expression with immune cell infiltration in GBM using TIMER: (A) CD274, (B) CTLA4, (C) HAVCR2, (D) LAG3, (E) TIGIT, (F) PDCD1, (G) PDCD1LG2, and (H) SIGLEC15. Figure S6: correlation of immune checkpoint gene expression with immune cell infiltration in LGG using TIMER: (A) CD274, (B) CTLA4, (C) HAVCR2, (D) LAG3, (E) TIGIT, (F) PDCD1, (G) PDCD1LG2, and (H) SIGLEC15.
