# Supplementary material for: Identification of Potential Immune Checkpoint Inhibitor Targets in Gliomas via Bioinformatic Analyses
Source: Biomed Res Int. 2022 Feb 14;2022:1734847. doi: 10.1155/2022/1734847 (PMC8860561; doi:10.1155/2022/1734847)

Supplementary File

Figure S1. The expression of immune checkpoint genes correlate with age and gender: (A) CD274, (B) CTLA4, (C) HAVCR2, (D) LAG3, (E) TIGIT, (F) PDCD1, (G) PDCD1LG2, and (H) SIGLEC15.

Figure1 A

CD274

A

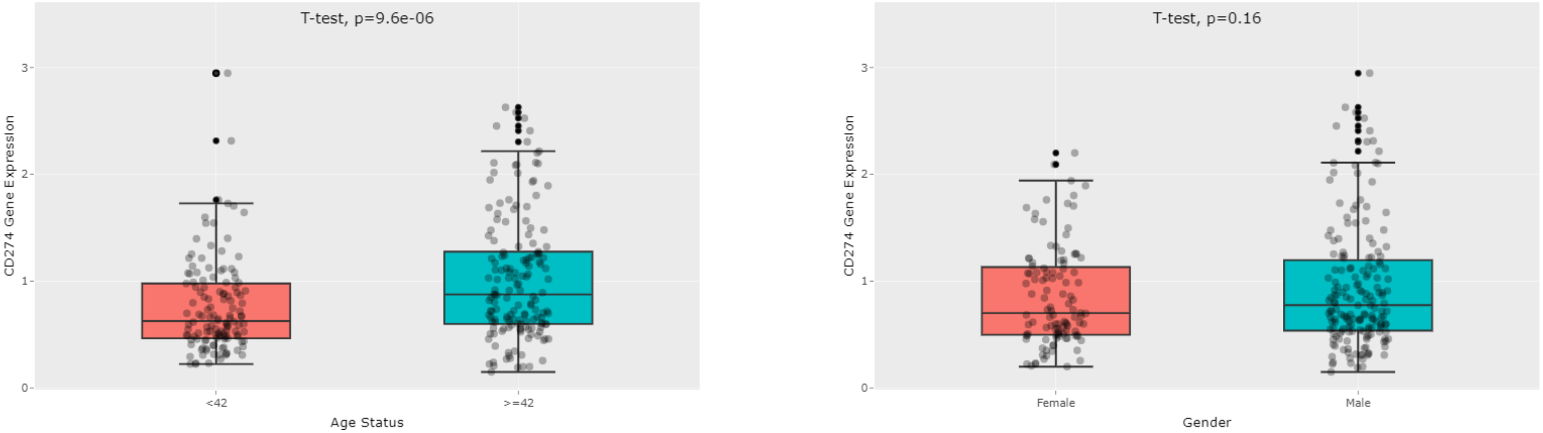

CTLA4

B

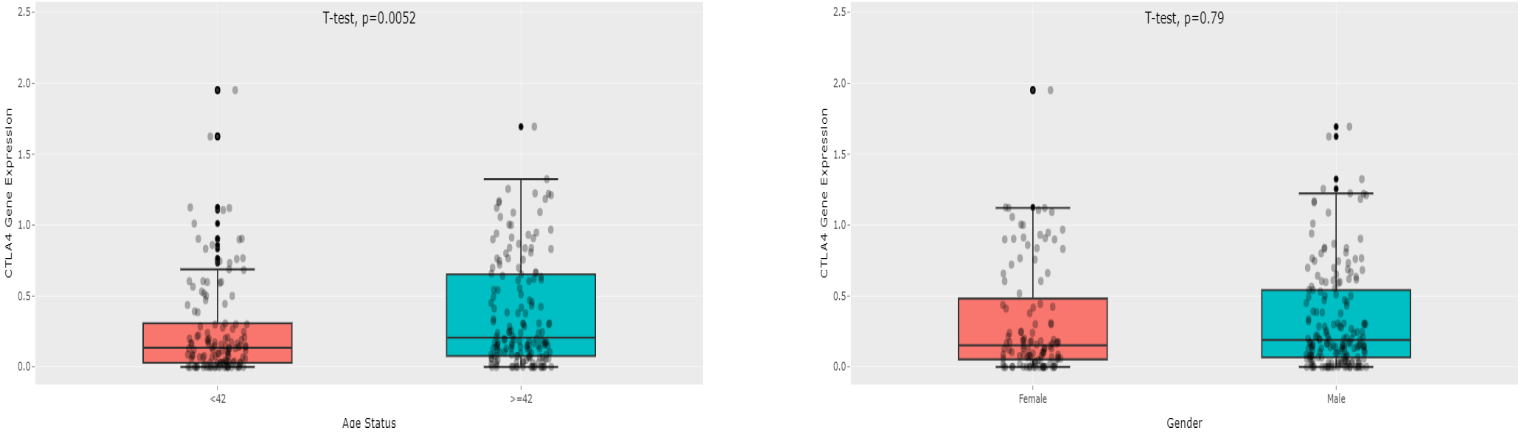

Figure1 B

HAVCR2

C

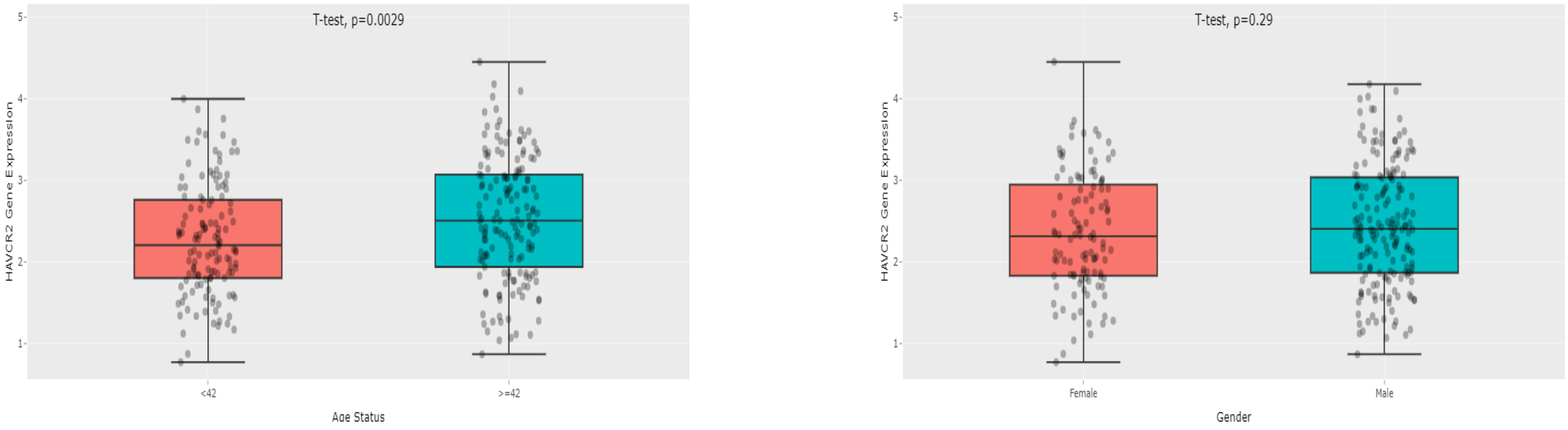

LAG3

D

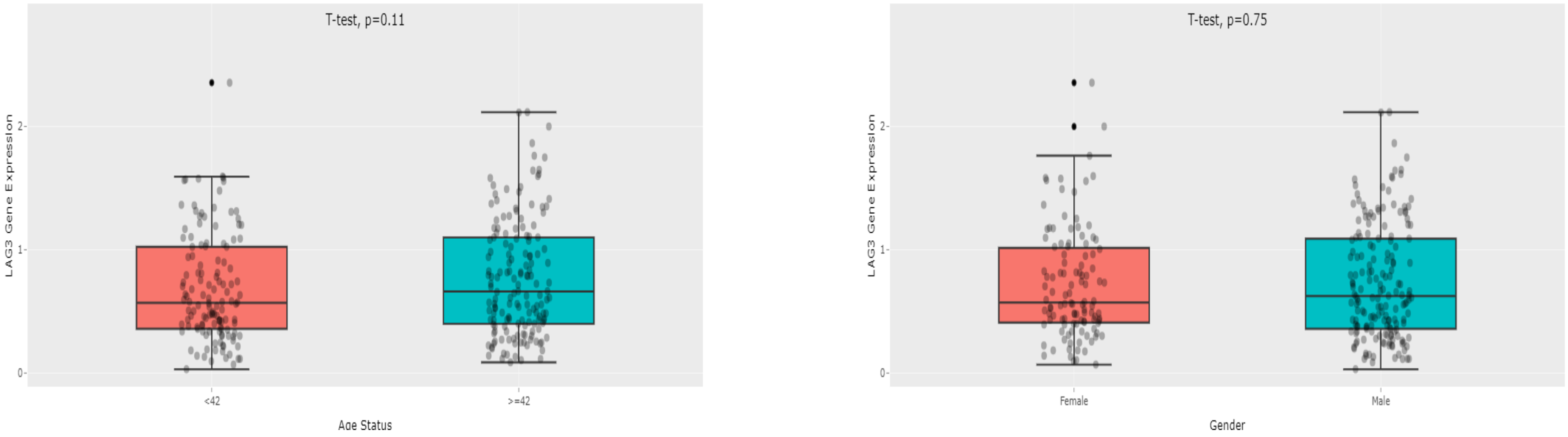

Figure1 C

TIGIT

E

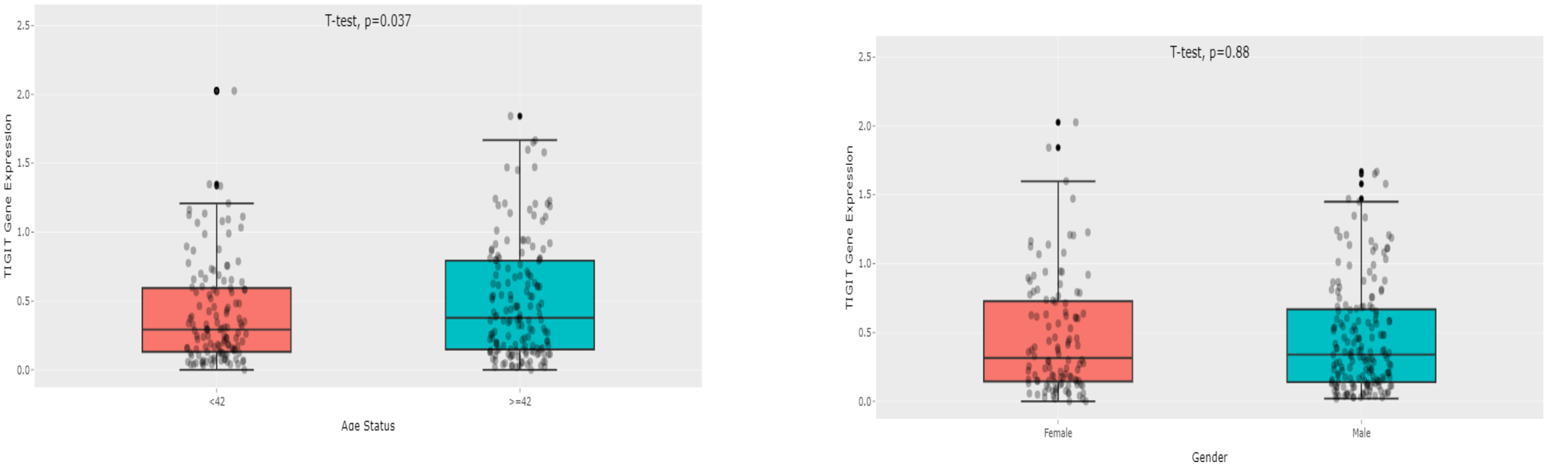

PDCD1

F

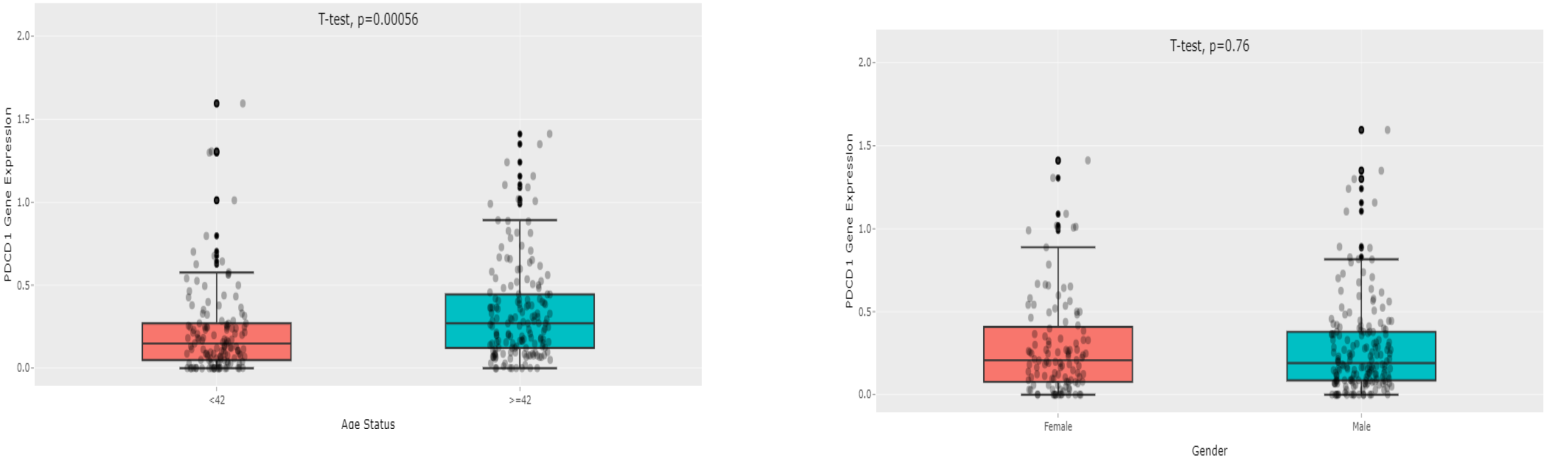

Figure1 D

PDCD1LG2

G

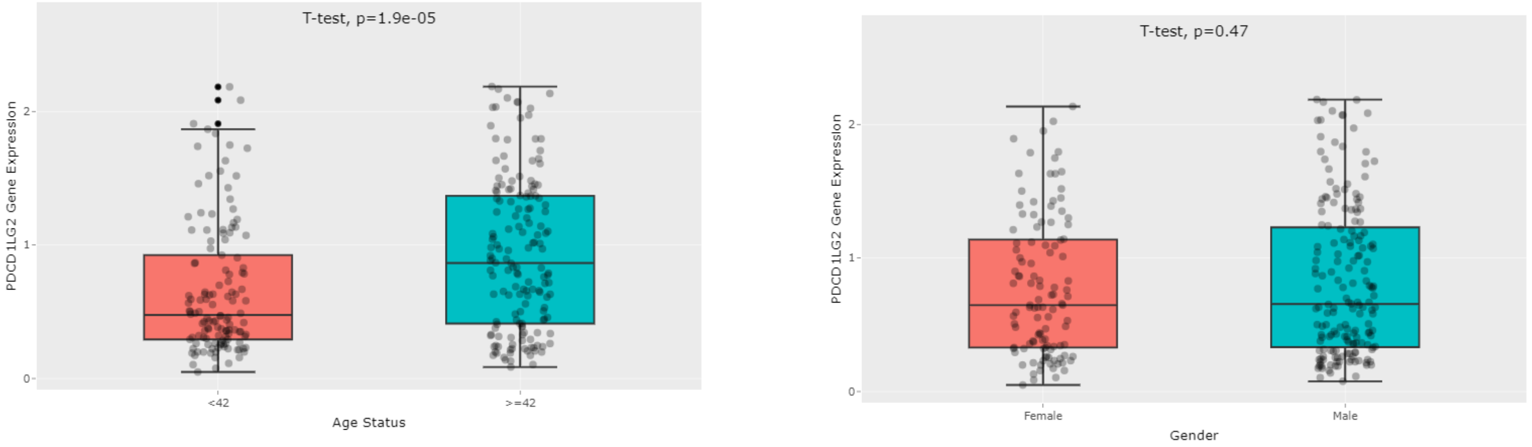

SIGLEC15

H

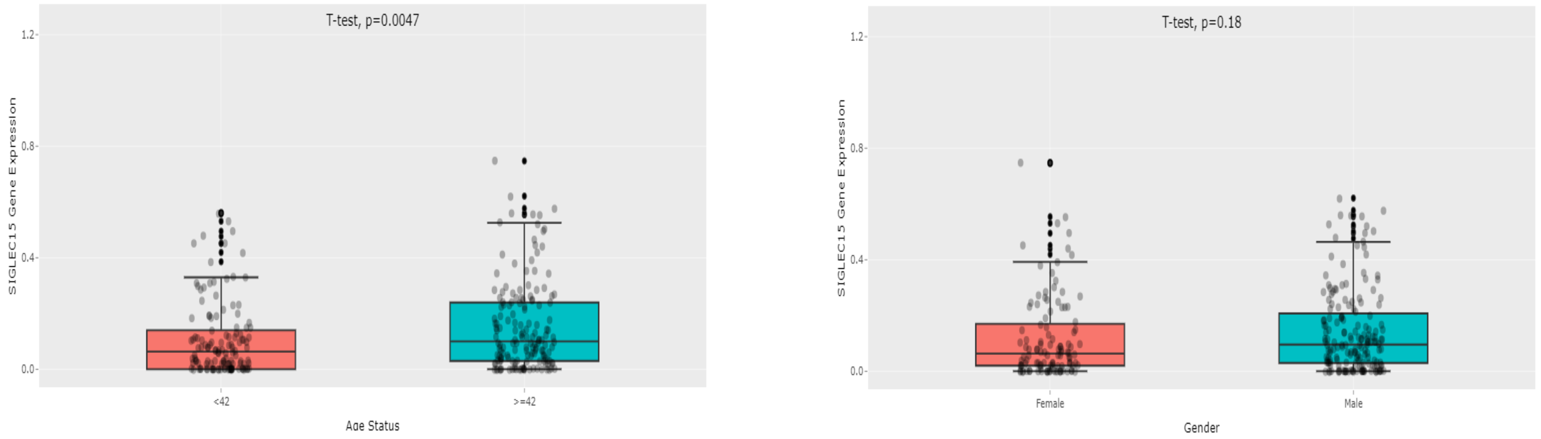

Figure S2. Kaplan-Meier overall survival curves of the immune checkpoint genes in glioma: (A) CD274, (B) CTLA4, (C) LAG3, (D) TIGIT, (E) PDCD1, (F) PDCD1LG2, and (G) SIGLEC15.

Figure2 A

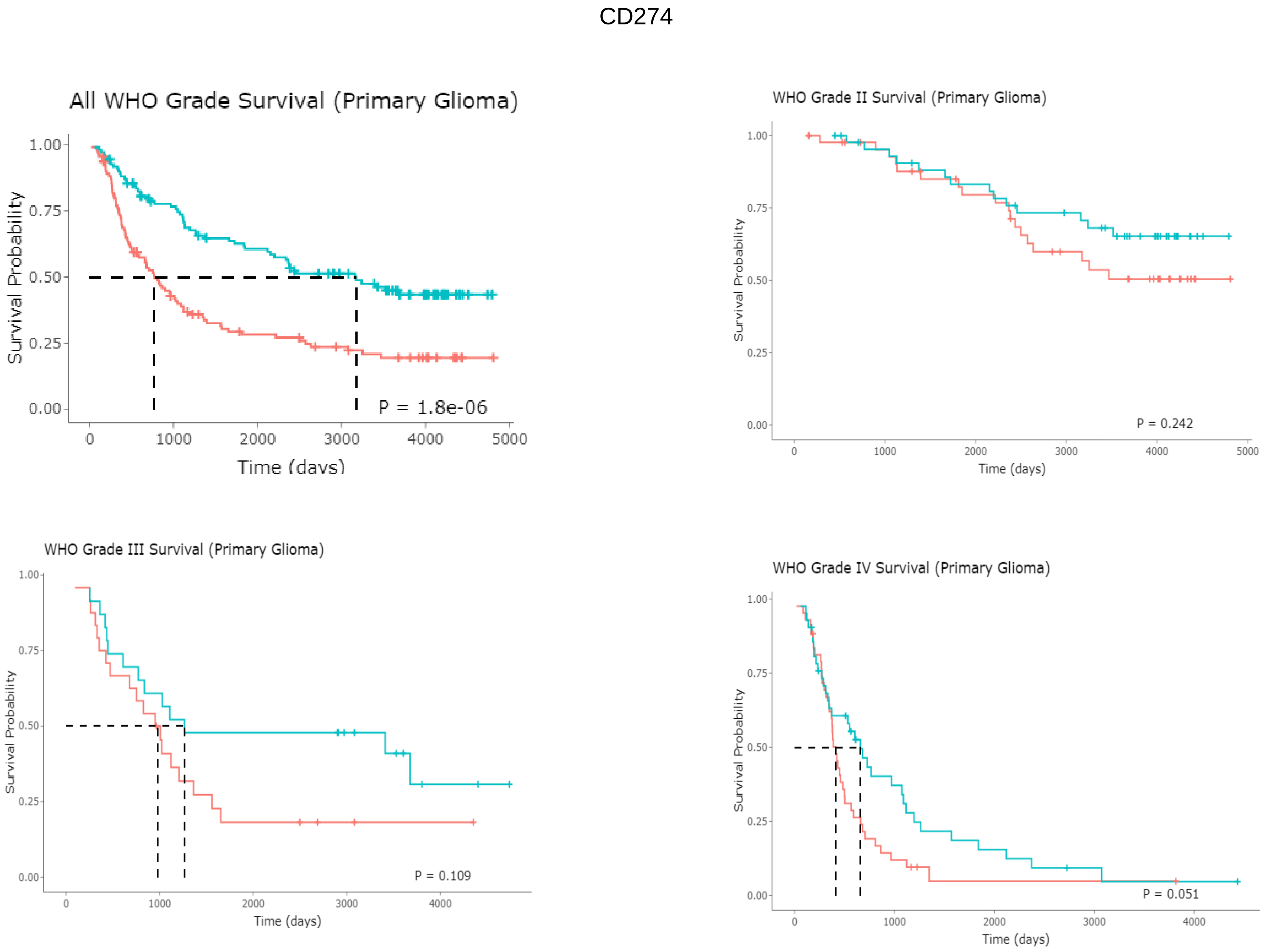

CD274
All WHO Grade Survival (Primary Glioma)
Survival Probability
1.00
0.75
0.50
0.25
0.00
0
1000
2000
3000
4000
5000
Time (days)
P = 1.8e-06
WHO Grade II Survival (Primary Glioma)
Survival Probability
Time (days)
P = 0.242
WHO Grade III Survival (Primary Glioma)
Survival Probability
Time (days)
P = 0.109
WHO Grade IV Survival (Primary Glioma)
Survival Probability
Time (days)
P = 0.051

Figure2 B

CTLA4

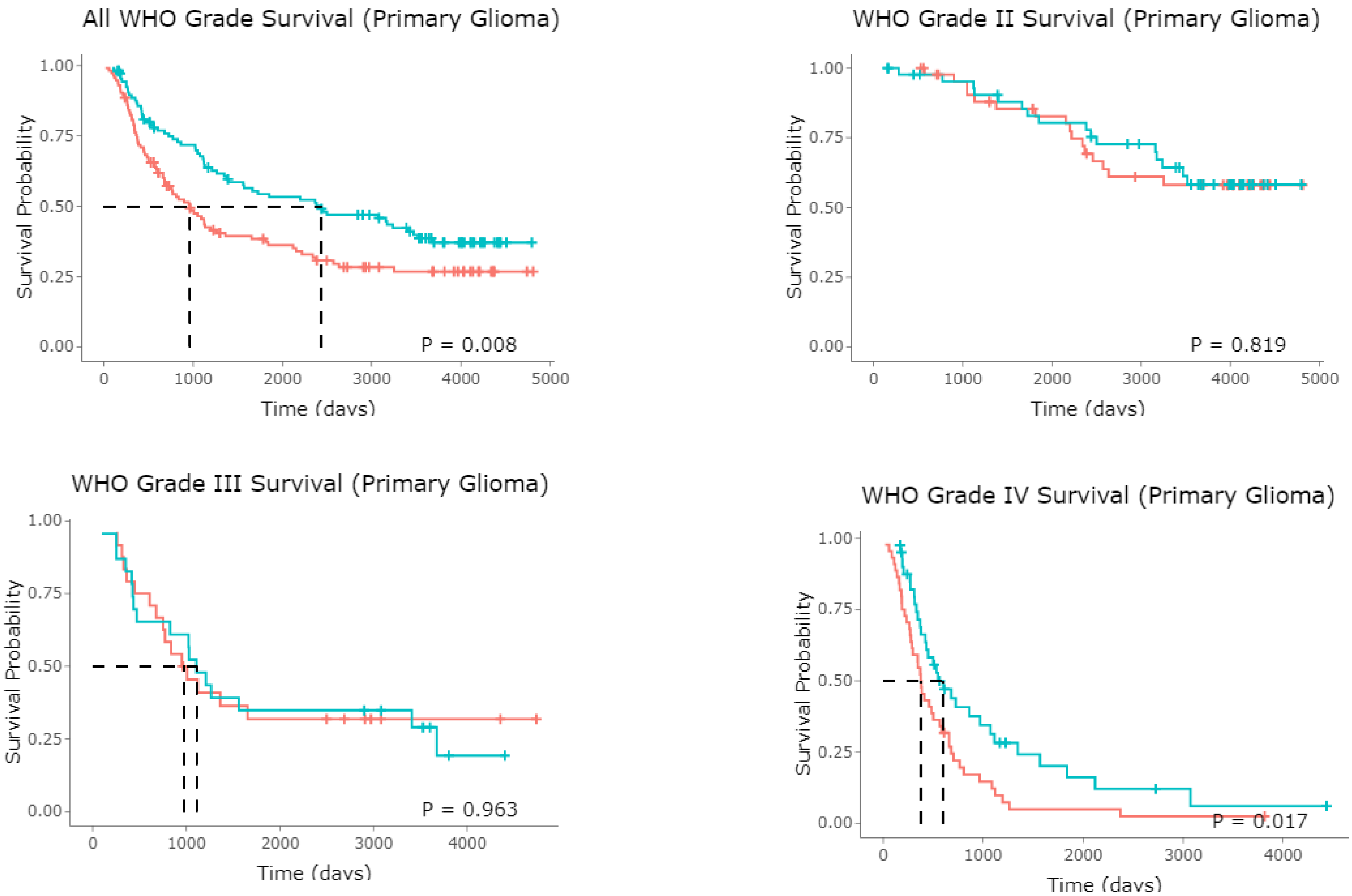

Figure2 C

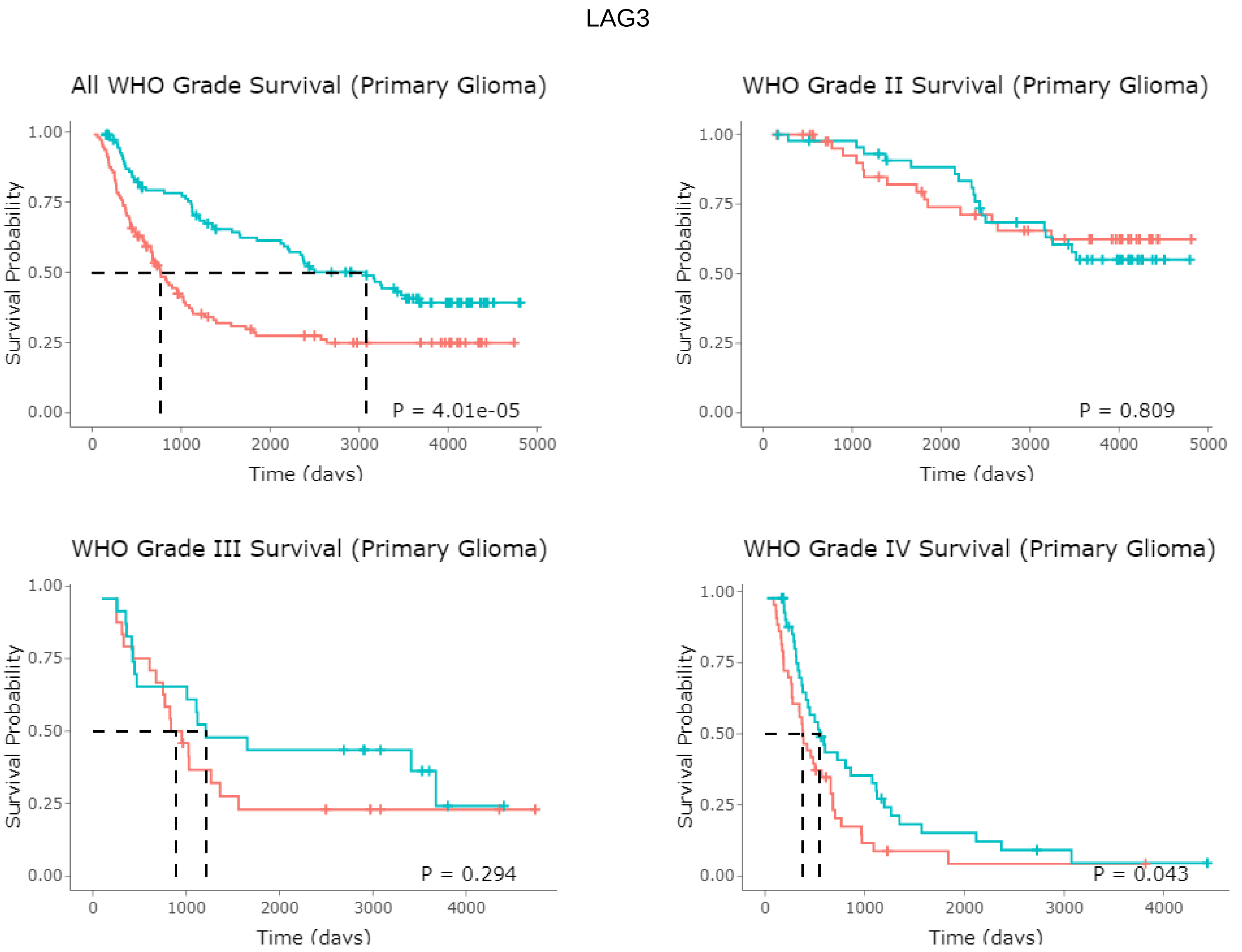
LAG3
All WHO Grade Survival (Primary Glioma)
WHO Grade II Survival (Primary Glioma)
WHO Grade III Survival (Primary Glioma)
WHO Grade IV Survival (Primary Glioma)
Survival Probability
Time (days)
P = 4.01e-05
P = 0.809
P = 0.294
P = 0.043

Figure2 D

TIGIT

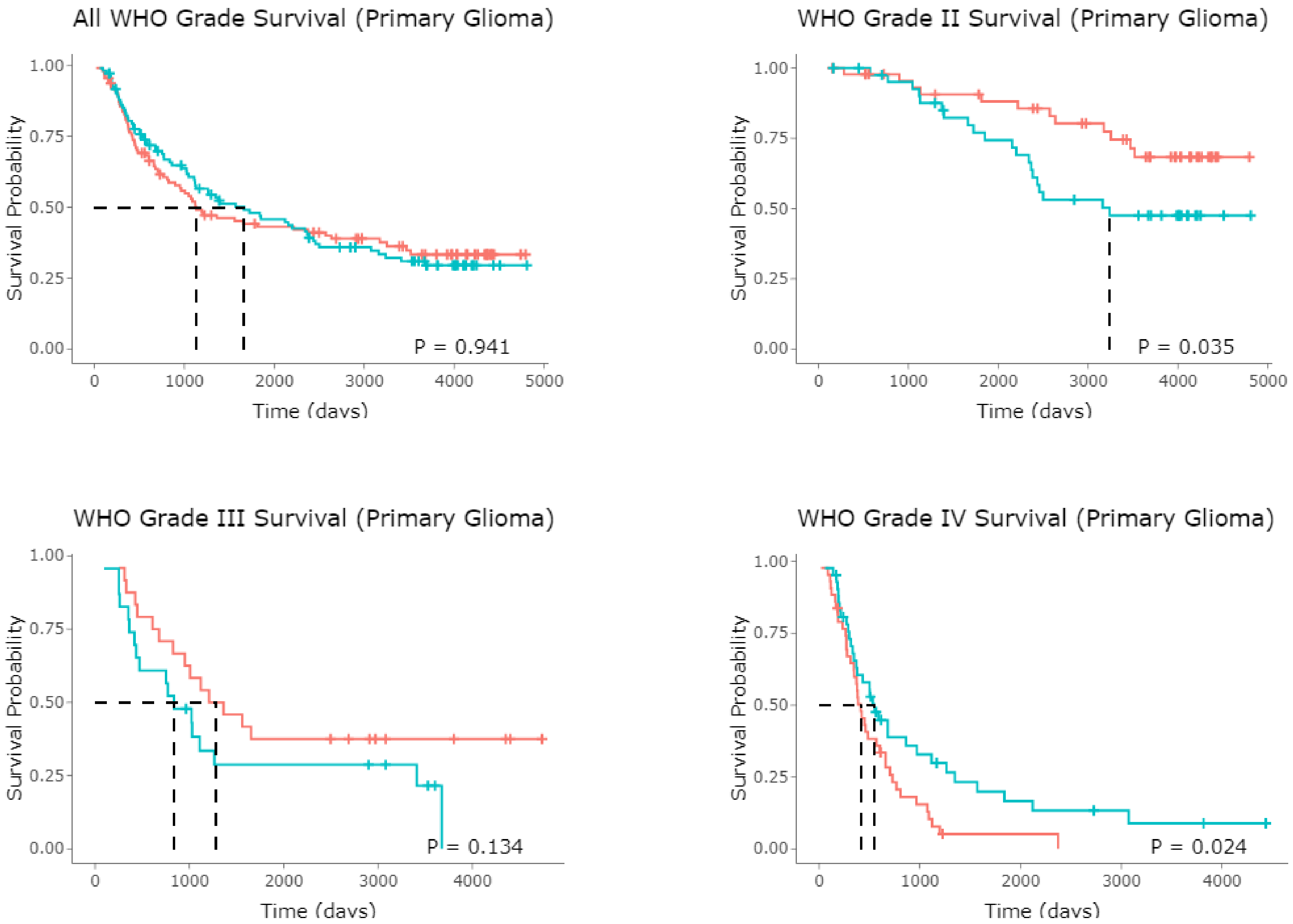

Figure2 E

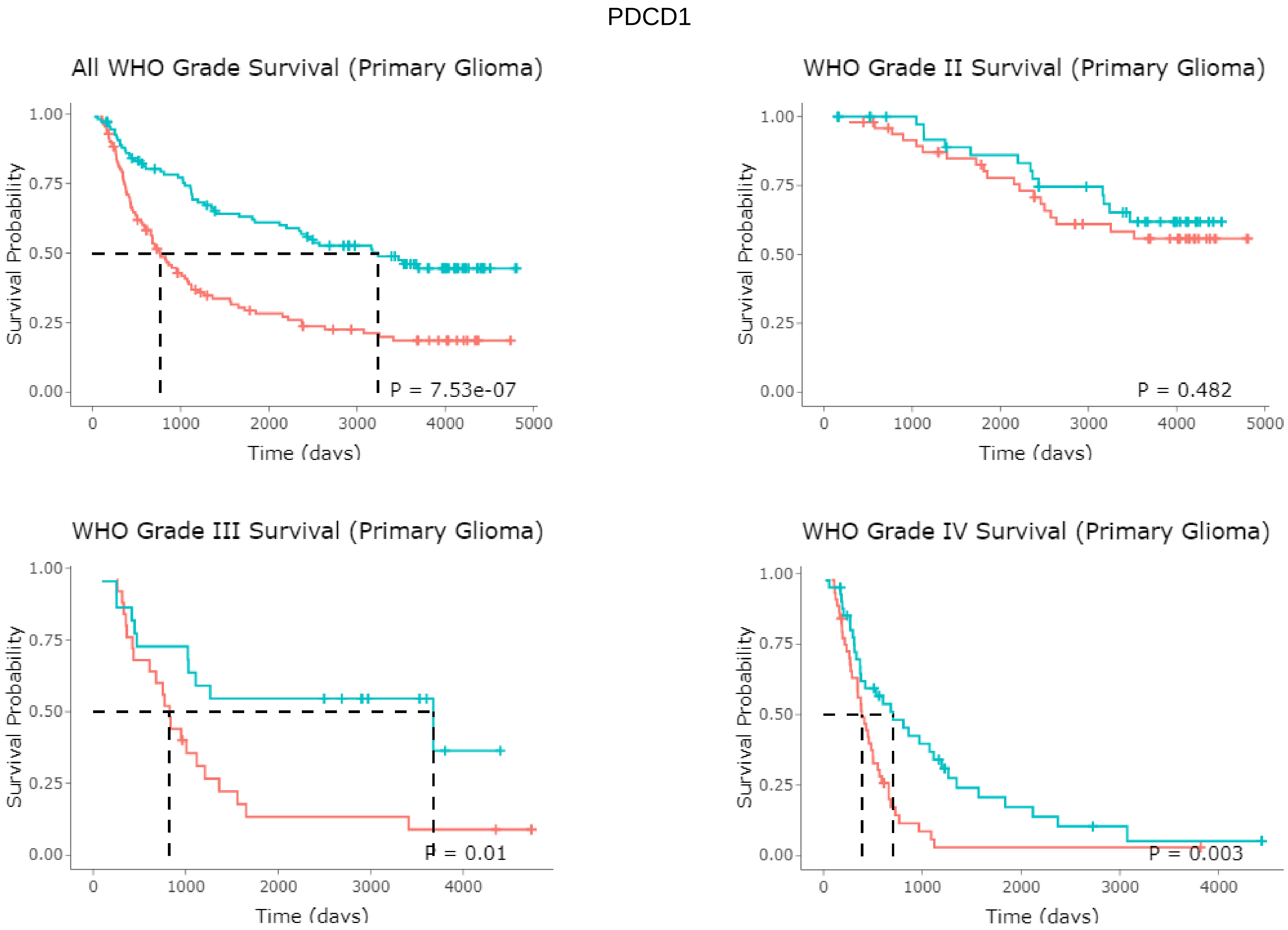
PDCD1
All WHO Grade Survival (Primary Glioma)
Survival Probability
1.00
0.75
0.50
0.25
0.00
0
1000
2000
3000
4000
5000
Time (days)
P = 7.53e-07
WHO Grade II Survival (Primary Glioma)
Survival Probability
1.00
0.75
0.50
0.25
0.00
0
1000
2000
3000
4000
5000
Time (days)
P = 0.482
WHO Grade III Survival (Primary Glioma)
Survival Probability
1.00
0.75
0.50
0.25
0.00
0
1000
2000
3000
4000
Time (days)
P = 0.01
WHO Grade IV Survival (Primary Glioma)
Survival Probability
1.00
0.75
0.50
0.25
0.00
0
1000
2000
3000
4000
Time (days)
P = 0.003

Figure2 F

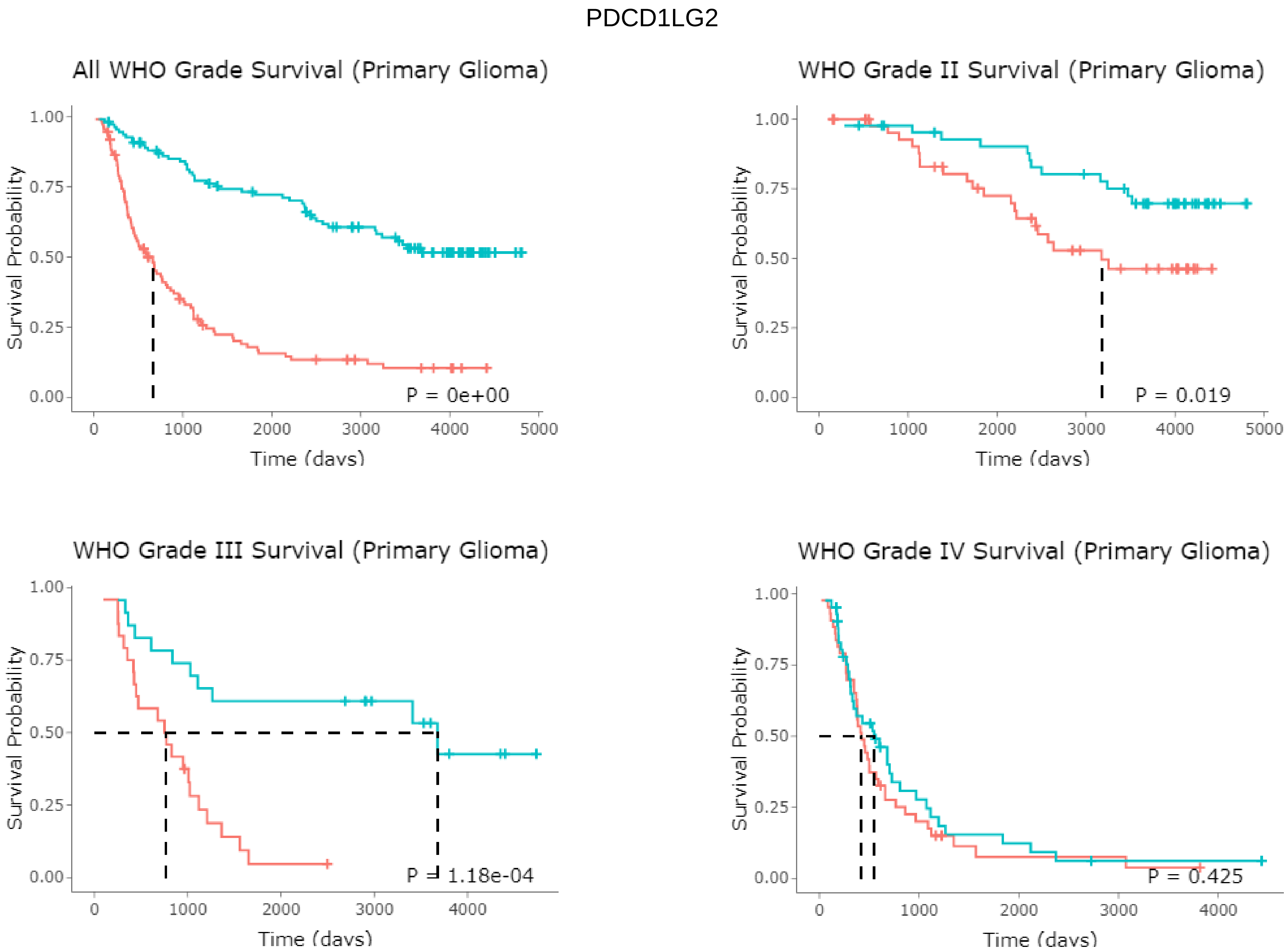
PDCD1LG2
All WHO Grade Survival (Primary Glioma)
WHO Grade II Survival (Primary Glioma)
WHO Grade III Survival (Primary Glioma)
WHO Grade IV Survival (Primary Glioma)
Survival Probability
Time (days)
P = 0e+00
P = 0.019
P = 1.18e-04
P = 0.425

Figure2 G

SIGLEC15

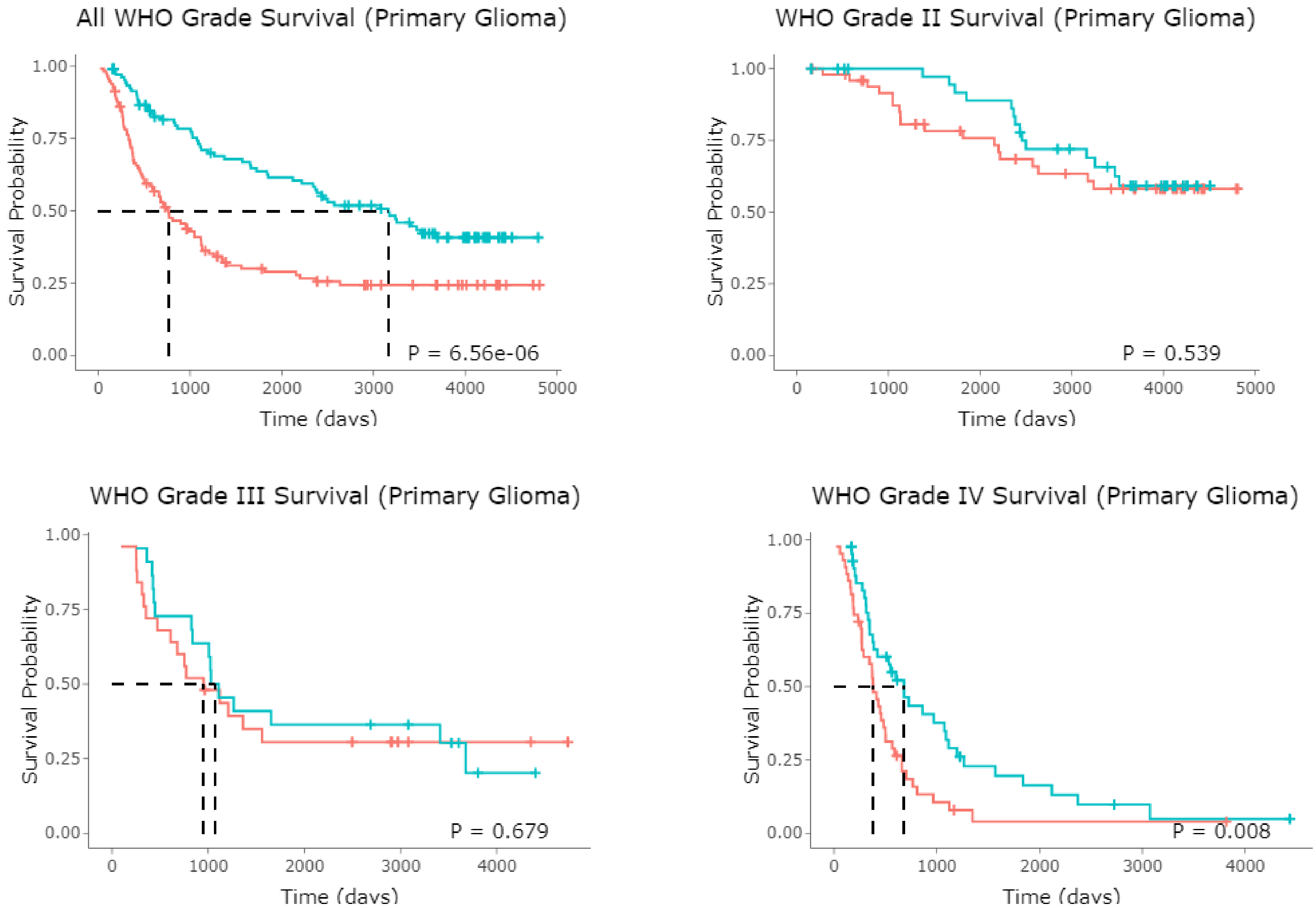

Figure S3. Correlation between the eight immune checkpoint genes: (A-B) GBM, (C-D) LGG.

Figure3 A

GBM

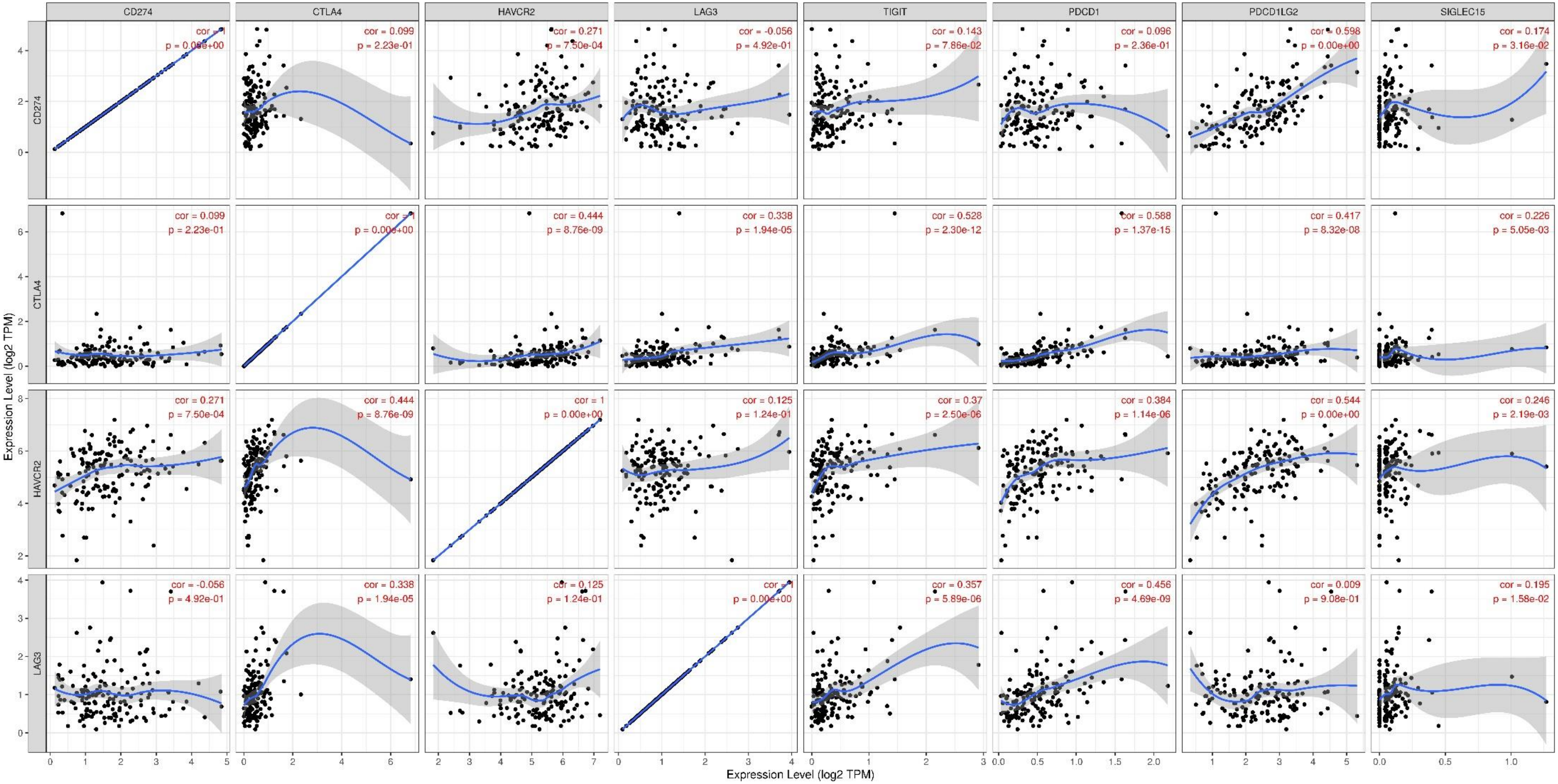

Figure3 B

GBM

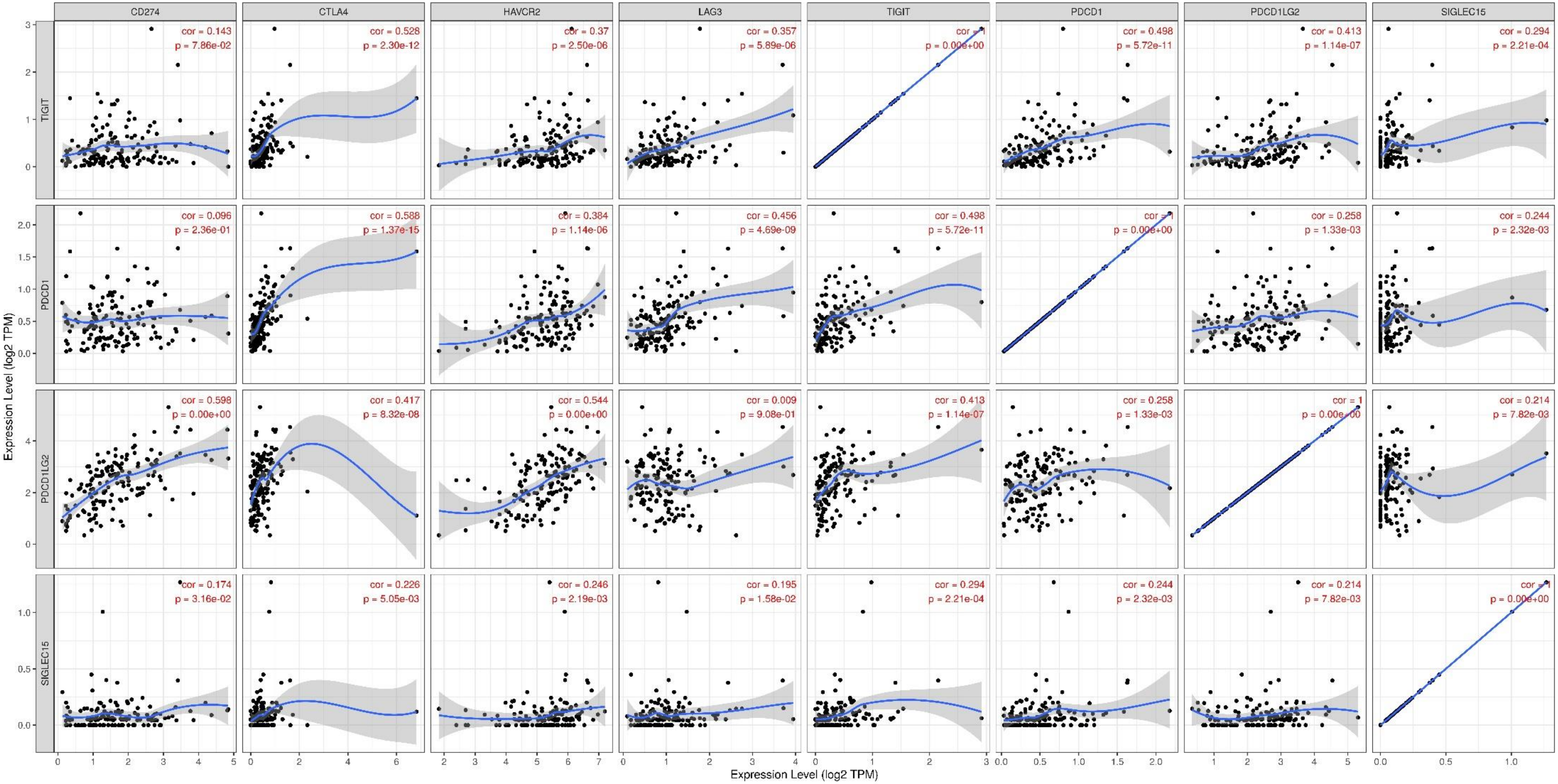

Figure3 C

LGG

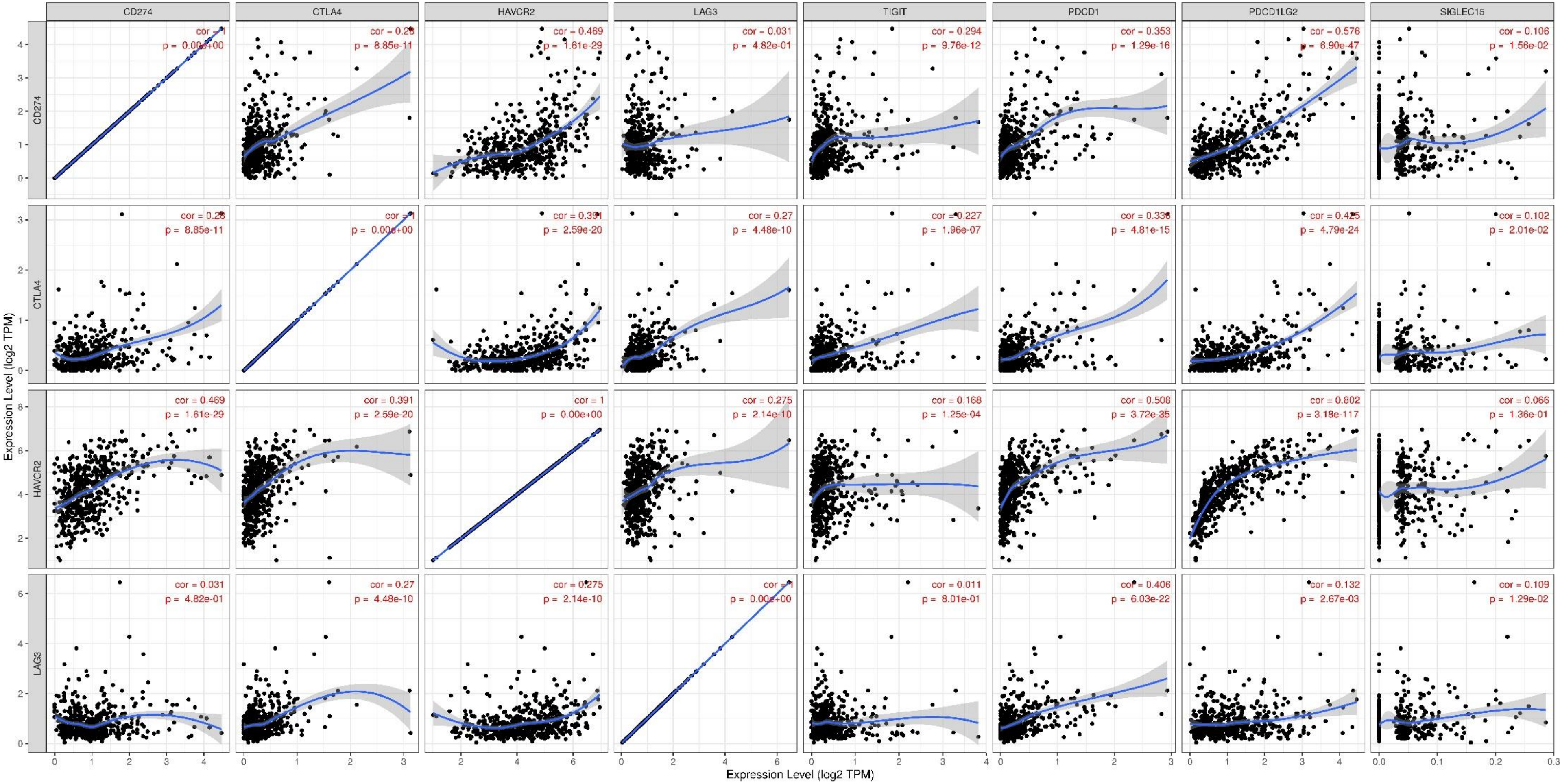

Figure3 D

LGG

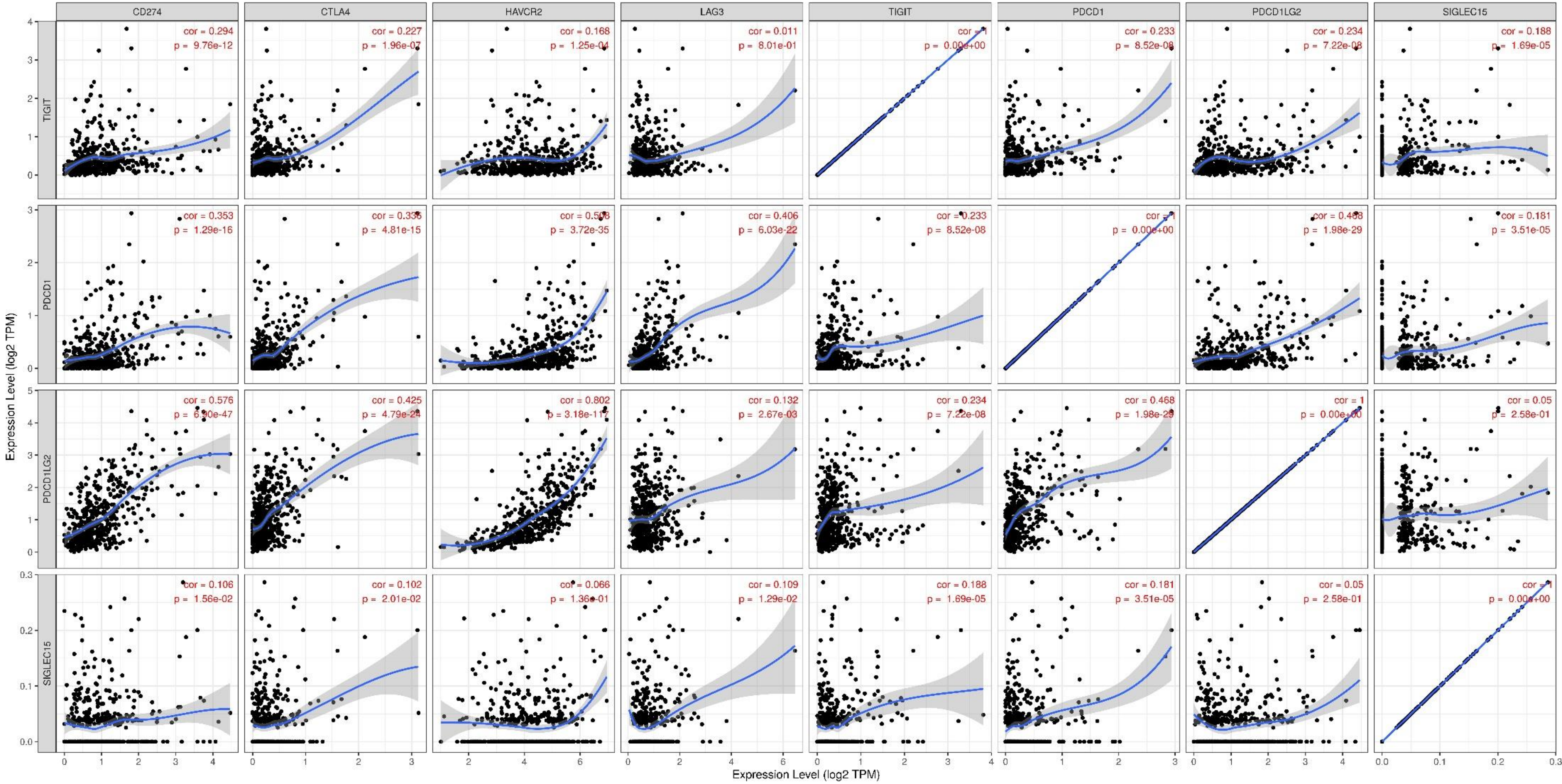

Figure S4. Heatmaps showing the correlation between HAVCR2 gene expression and tumor-immune system interactions across all cancers in TCGA. Correlation between HAVCR2 gene expression and (A) tumor-infiltrating lymphocytes (TILs), (B) immune inhibitors, (C) immune stimulators, (D) MHC molecules, (E) chemokines, and (F) receptors.

Figure4

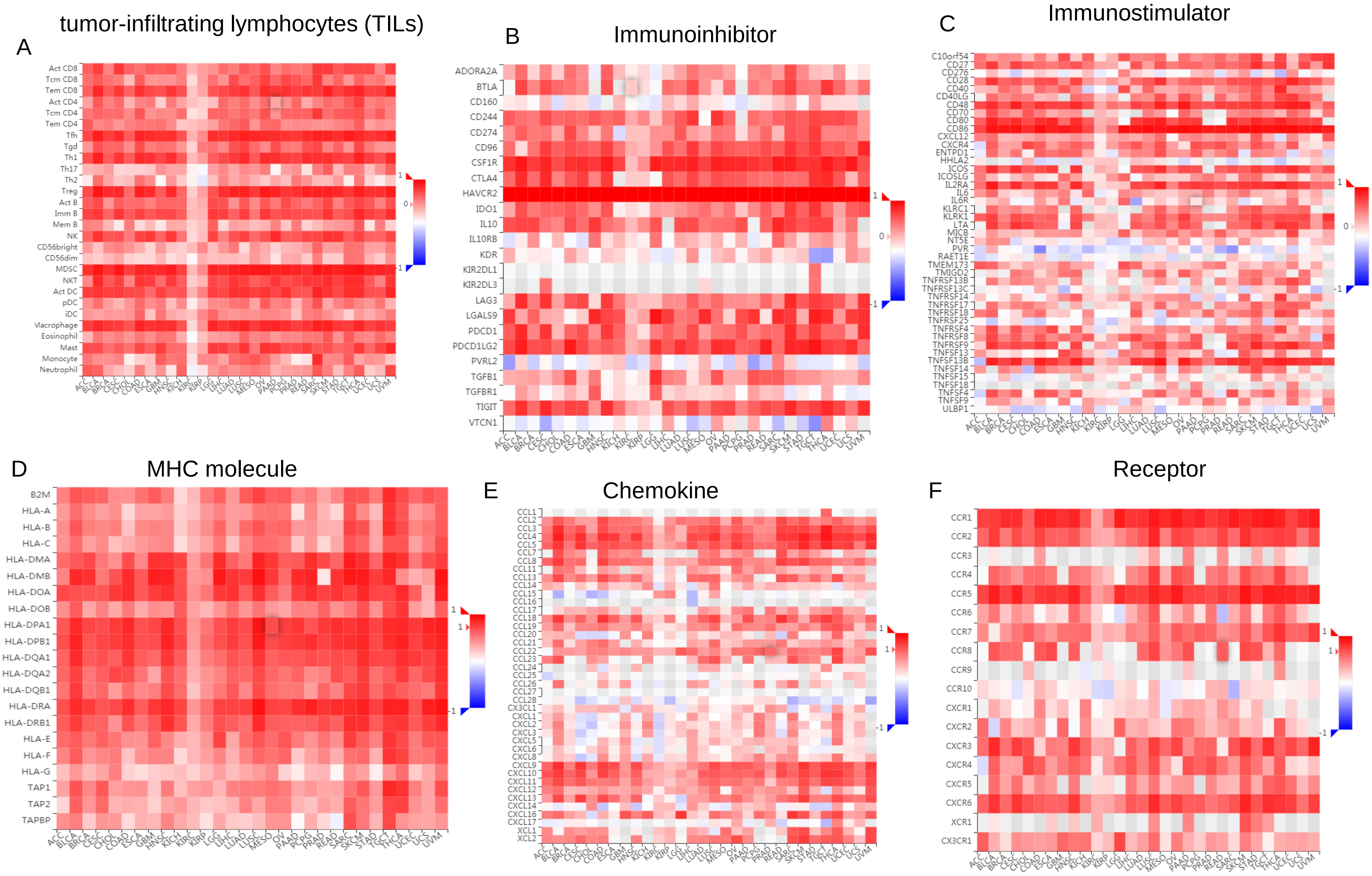
A
tumor-infiltrating lymphocytes (TILs)
Act CD8
Tcm CD8
Tem CD8
Act CD4
Tcm CD4
Tem CD4
Tfh
Tgd
Th1
Th17
Th2
Treg
Act B
Imm B
Mem B
NK
CD56bright
CD56dim
MDSC
NKT
Act DC
pDC
iDC
Macrophage
Eosinophil
Mast
Monocyte
Neutrophil
ACC BLCA BRCA CESC CHOL COAD ESCA GBM HNSC KICH KIRC KIRP LGG LIHC LUAD LUSC MESO OV PAAD PCPG PRAD READ SARC SKCM STAD TGCT THCA UCEC UCS UVM
1
0
-1
B
Immunoinhibitor
ADORA2A
BTLA
CD160
CD244
CD274
CD96
CSF1R
CTLA4
HAVCR2
IDO1
IL10
IL10RB
KDR
KIR2DL1
KIR2DL3
LAG3
LGALS9
PDCD1
PDCD1LG2
PVRL2
TGFB1
TGFBR1
TIGIT
VTCN1
ACC BLCA BRCA CESC CHOL COAD ESCA GBM HNSC KICH KIRC KIRP LGG LIHC LUAD LUSC MESO OV PAAD PCPG PRAD READ SARC SKCM STAD TGCT THCA UCEC UCS UVM
1
0
-1
C
Immunostimulator
C10orf54
CD27
CD276
CD28
CD40
CD40LG
CD48
CD70
CD80
CD86
CXCL12
CXCR4
ENTPD1
HHLA2
ICOS
ICOSLG
IL2RA
IL6
IL6R
KLRC1
KLRK1
LTA
MICB
NT5E
PVR
RAET1E
TMEM173
TMIGD2
TNFRSF13B
TNFRSF13C
TNFRSF14
TNFRSF17
TNFRSF18
TNFRSF25
TNFRSF4
TNFRSF8
TNFRSF9
TNFSF13
TNFSF13B
TNFSF14
TNFSF15
TNFSF18
TNFSF4
TNFSF9
ULBP1
ACC BLCA BRCA CESC CHOL COAD ESCA GBM HNSC KICH KIRC KIRP LGG LIHC LUAD LUSC MESO OV PAAD PCPG PRAD READ SARC SKCM STAD TGCT THCA UCEC UCS UVM
1
0
-1
D
MHC molecule
B2M
HLA-A
HLA-B
HLA-C
HLA-DMA
HLA-DMB
HLA-DOA
HLA-DOB
HLA-DPA1
HLA-DPB1
HLA-DQA1
HLA-DQA2
HLA-DQB1
HLA-DRA
HLA-DRB1
HLA-E
HLA-F
HLA-G
TAP1
TAP2
TAPBP
ACC BLCA BRCA CESC CHOL COAD ESCA GBM HNSC KICH KIRC KIRP LGG LIHC LUAD LUSC MESO OV PAAD PCPG PRAD READ SARC SKCM STAD TGCT THCA UCEC UCS UVM
1
1
-1
E
Chemokine
CCL1
CCL2
CCL3
CCL4
CCL5
CCL7
CCL8
CCL11
CCL13
CCL14
CCL15
CCL16
CCL17
CCL18
CCL19
CCL20
CCL21
CCL22
CCL23
CCL24
CCL25
CCL26
CCL27
CCL28
CX3CL1
CXCL1
CXCL2
CXCL3
CXCL5
CXCL6
CXCL8
CXCL9
CXCL10
CXCL11
CXCL12
CXCL13
CXCL14
CXCL16
CXCL17
XCL1
XCL2
ACC BLCA BRCA CESC CHOL COAD ESCA GBM HNSC KICH KIRC KIRP LGG LIHC LUAD LUSC MESO OV PAAD PCPG PRAD READ SARC SKCM STAD TGCT THCA UCEC UCS UVM
1
1
-1
F
Receptor
CCR1
CCR2
CCR3
CCR4
CCR5
CCR6
CCR7
CCR8
CCR9
CCR10
CXCR1
CXCR2
CXCR3
CXCR4
CXCR5
CXCR6
XCR1
CX3CR1
ACC BLCA BRCA CESC CHOL COAD ESCA GBM HNSC KICH KIRC KIRP LGG LIHC LUAD LUSC MESO OV PAAD PCPG PRAD READ SARC SKCM STAD TGCT THCA UCEC UCS UVM
1
1
-1

Figure S5. Correlation of immune checkpoint gene expression with immune cell infiltration in GBM using TIMER: (A) CD274, (B) CTLA4, (C) HAVCR2, (D) LAG3, (E) TIGIT, (F) PDCD1, (G) PDCD1LG2, and (H) SIGLEC15.

Figure5-1

GBM

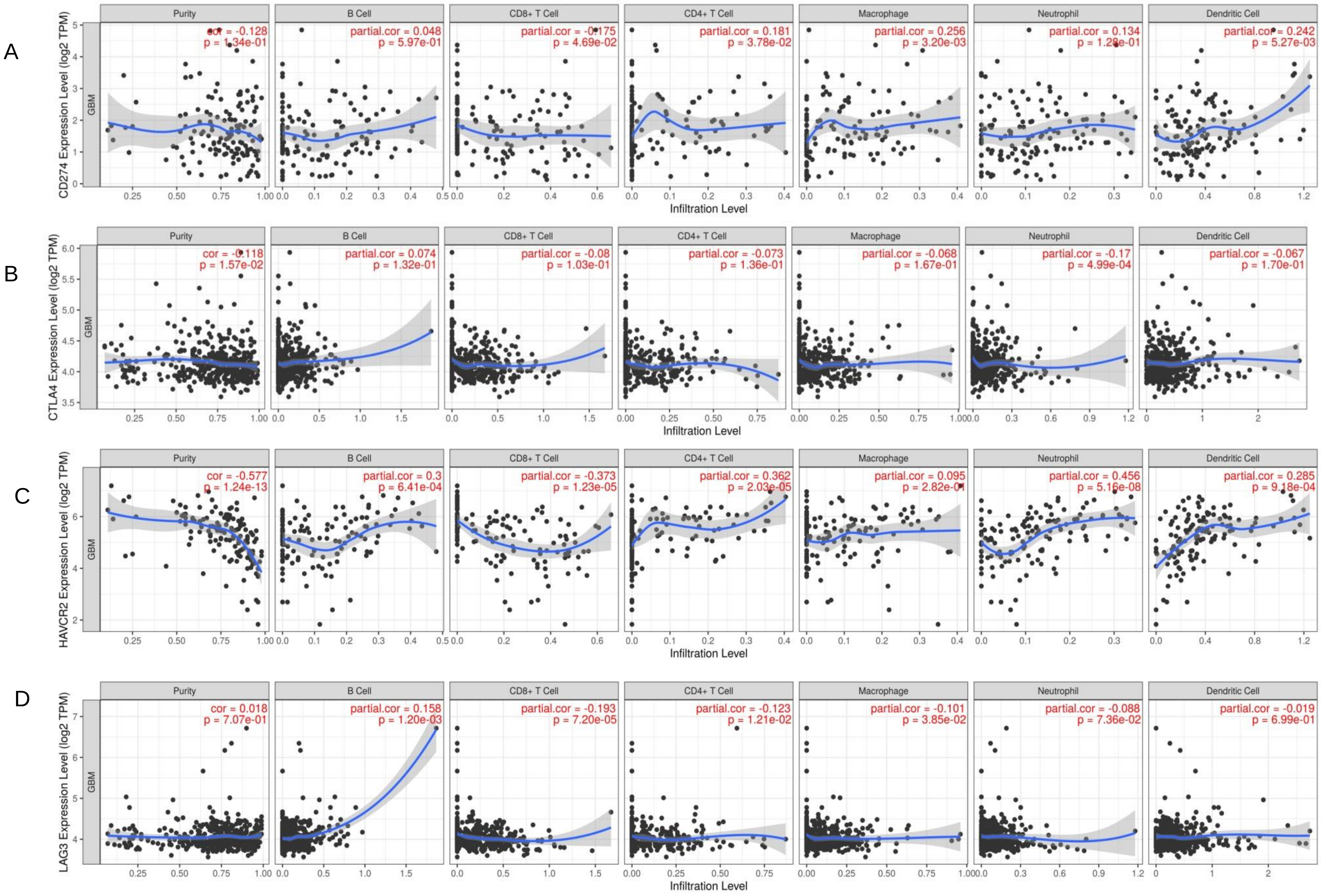

Figure5-2

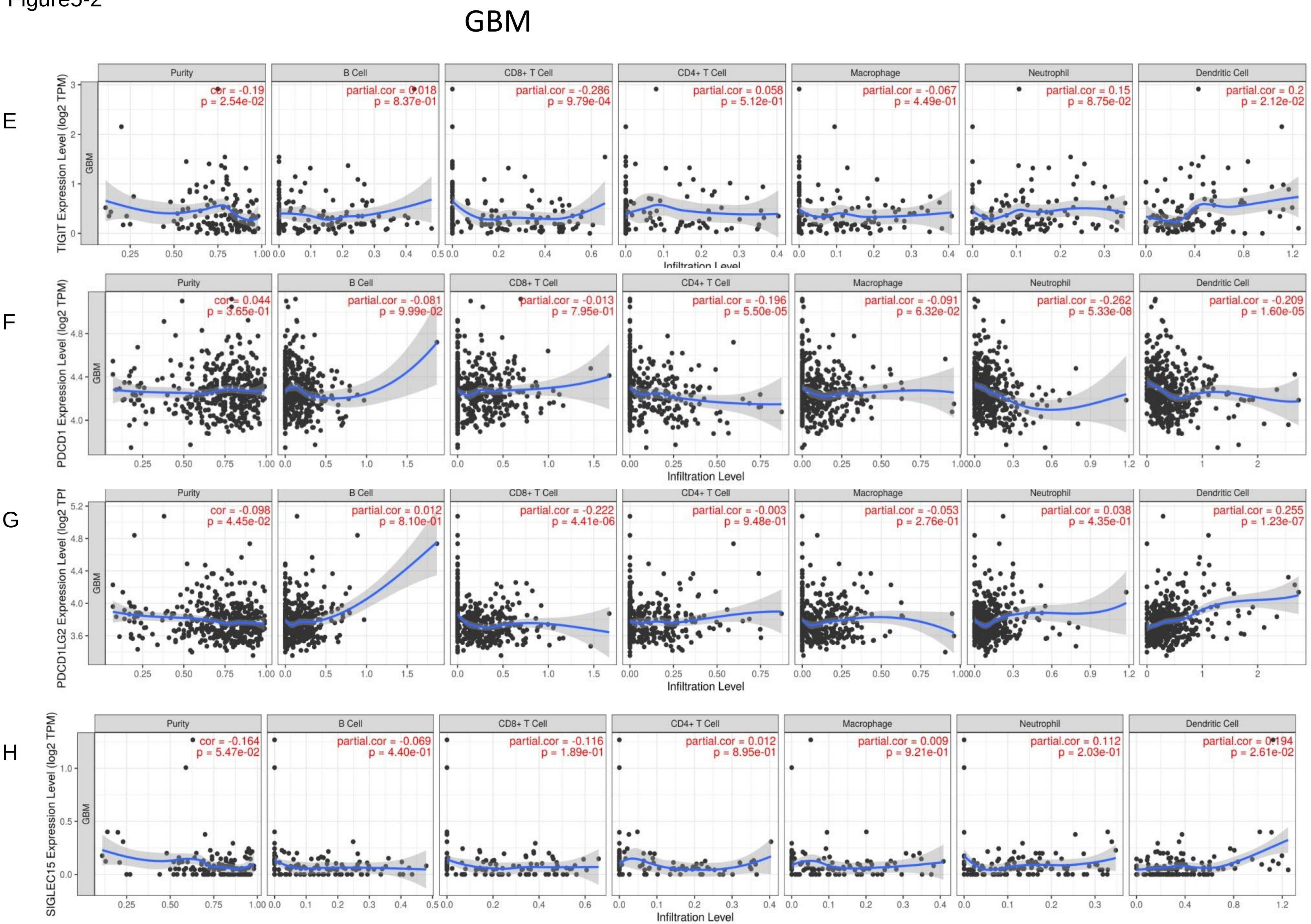
GBM
E
F
G
H
TIGIT Expression Level (log2 TPM)
PDCD1 Expression Level (log2 TPM)
PDCD1LG2 Expression Level (log2 TPM)
SIGLEC15 Expression Level (log2 TPM)
Purity
B Cell
CD8+ T Cell
CD4+ T Cell
Macrophage
Neutrophil
Dendritic Cell
Infiltration Level
cor = -0.19 p = 2.54e-02
partial.cor = 0.018 p = 8.37e-01
partial.cor = -0.286 p = 9.79e-04
partial.cor = 0.058 p = 5.12e-01
partial.cor = -0.067 p = 4.49e-01
partial.cor = 0.15 p = 8.75e-02
partial.cor = 0.2 p = 2.12e-02
cor = 0.044 p = 3.65e-01
partial.cor = -0.081 p = 9.99e-02
partial.cor = -0.013 p = 7.95e-01
partial.cor = -0.196 p = 5.50e-05
partial.cor = -0.091 p = 6.32e-02
partial.cor = -0.262 p = 5.33e-08
partial.cor = -0.209 p = 1.60e-05
cor = -0.098 p = 4.45e-02
partial.cor = 0.012 p = 8.10e-01
partial.cor = -0.222 p = 4.41e-06
partial.cor = -0.003 p = 9.48e-01
partial.cor = -0.053 p = 2.76e-01
partial.cor = 0.038 p = 4.35e-01
partial.cor = 0.255 p = 1.23e-07
cor = -0.164 p = 5.47e-02
partial.cor = -0.069 p = 4.40e-01
partial.cor = -0.116 p = 1.89e-01
partial.cor = 0.012 p = 8.95e-01
partial.cor = 0.009 p = 9.21e-01
partial.cor = 0.112 p = 2.03e-01
partial.cor = 0.194 p = 2.61e-02

Figure S6. Correlation of immune checkpoint gene expression with immune cell infiltration in LGG using TIMER: (A) CD274, (B) CTLA4, (C) HAVCR2, (D) LAG3, (E) TIGIT, (F) PDCD1, (G) PDCD1LG2, and (H) SIGLEC15.

Figure6-1

LGG

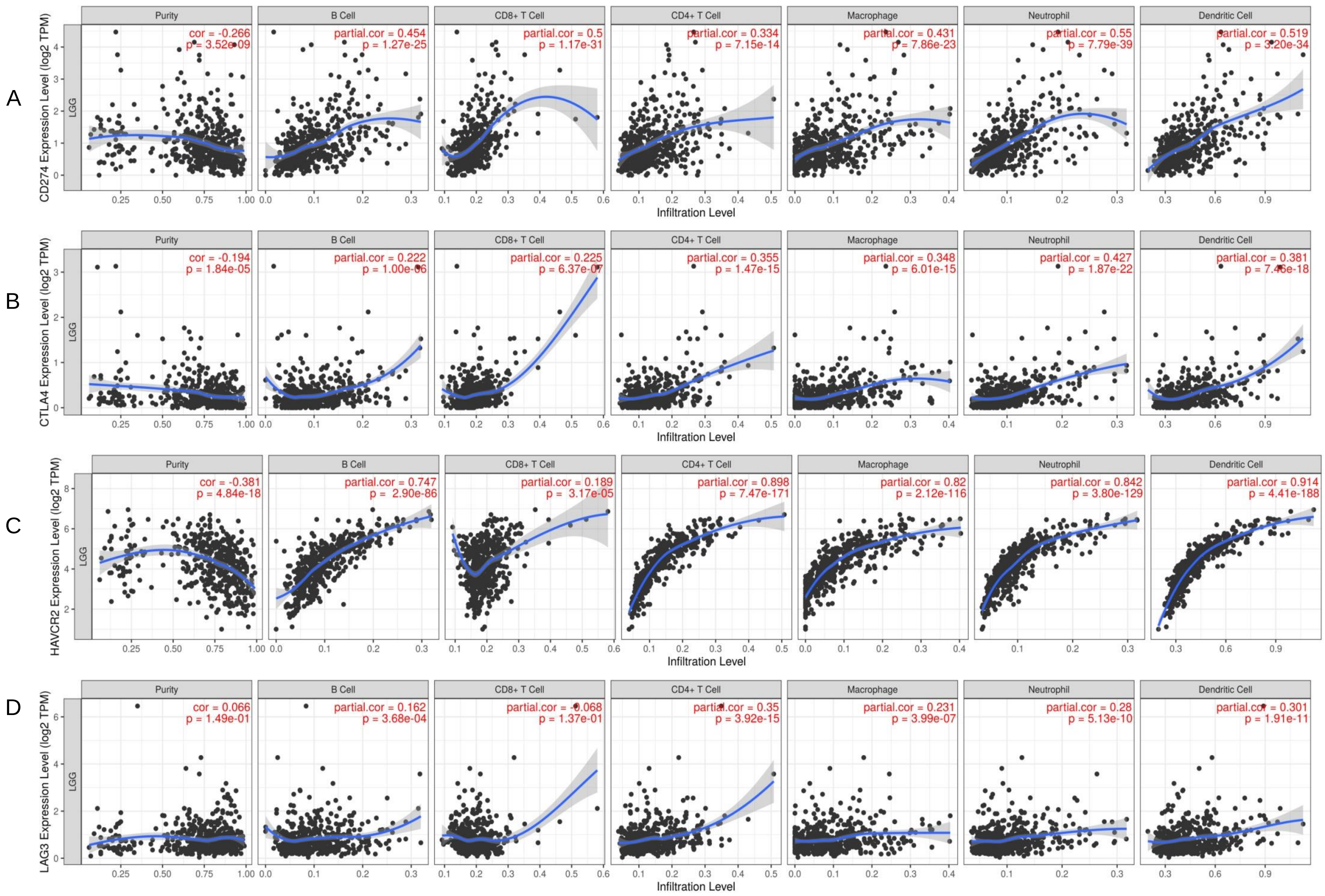

Figure6-2

LGG

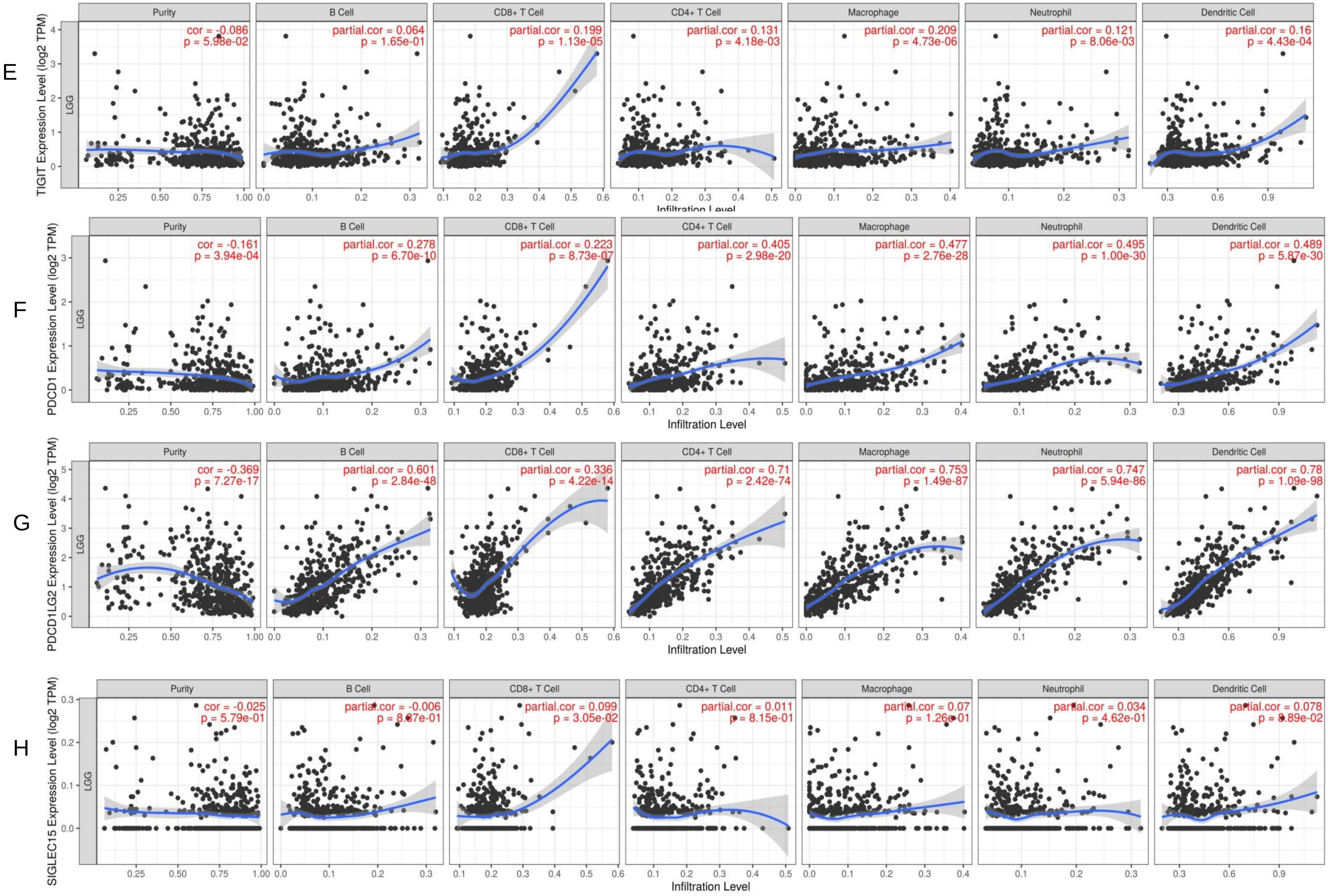

Supplement: Supplementary Materials — Figure S1: the expression of immune checkpoint genes correlates with age and gender: (A) CD274, (B) CTLA4, (C) HAVCR2, (D) LAG3, (E) TIGIT, (F) PDCD1, (G) PDCD1LG2, and (H) SIGLEC15. Figure S2: Kaplan-Meier overall survival curves of the immune checkpoint genes in glioma: (A) CD274, (B) CTLA4, (C) LAG3, (D) TIGIT, (E) PDCD1, (F) PDCD1LG2, and (G) SIGLEC15. Figure S3: Correlation between the eight immune checkpoint genes: (A-B) GBM and (C-D) LGG. Figure S4: heatmaps showing the correlation between HAVCR2 gene expression and tumor-immune system interactions across all cancers in TCGA. Correlation between HAVCR2 gene expression and (A) tumor-infiltrating lymphocytes (TILs), (B) immune inhibitors, (C) immune stimulators, (D) MHC molecules, (E) chemokines, and (F) receptors. Figure S5: correlation of immune checkpoint gene expression with immune cell infiltration in GBM using TIMER: (A) CD274, (B) CTLA4, (C) HAVCR2, (D) LAG3, (E) TIGIT, (F) PDCD1, (G) PDCD1LG2, and (H) SIGLEC15. Figure S6: correlation of immune checkpoint gene expression with immune cell infiltration in LGG using TIMER: (A) CD274, (B) CTLA4, (C) HAVCR2, (D) LAG3, (E) TIGIT, (F) PDCD1, (G) PDCD1LG2, and (H) SIGLEC15. [file 1734847.f1.pdf]
